# Fluorinated derivatives of pyridine-2,4-dicarboxylate are potent inhibitors of human 2-oxoglutarate dependent oxygenases

**DOI:** 10.1016/j.jfluchem.2021.109804

**Published:** 2021-07

**Authors:** Lennart Brewitz, Yu Nakashima, Anthony Tumber, Eidarus Salah, Christopher J. Schofield

**Affiliations:** a Chemistry Research Laboratory, University of Oxford, 12 Mansfield Road, OX1 3TA, Oxford, United Kingdom; b Present address: Institute of Natural Medicine, University of Toyama, 2630-Sugitani, 930-0194, Toyama, Japan

**Keywords:** 2-Oxoglutarate / α-ketoglutarate dependent oxygenase, Pyridine-2,4-dicarboxylic acid / 2,4-PDCA, JmjC lysine demethylase / KDM, Aspartate/asparagine-β-hydroxylase / AspH / BAH / HAAH, Ribosomal oxygenase 2 / RIOX2 / Mina53, Fluorinated hydroxylase inhibitor

## Abstract

2-Oxoglutarate (2OG) oxygenases have important roles in human biology and are validated medicinal chemistry targets. Improving the selectivity profile of broad-spectrum 2OG oxygenase inhibitors may help enable the identification of selective inhibitors for use in functional assignment work. We report the synthesis of F- and CF_3_-substituted derivatives of the broad-spectrum 2OG oxygenase inhibitor pyridine-2,4-dicarboxylate (2,4-PDCA). Their inhibition selectivity profile against selected functionally distinct human 2OG oxygenases was determined using mass spectrometry-based assays. F-substituted 2,4-PDCA derivatives efficiently inhibit the 2OG oxygenases aspartate/asparagine-β-hydroxylase (AspH) and the JmjC lysine-specific *N*^ε^-demethylase 4E (KDM4E); The F- and CF_3_-substituted 2,4-PDCA derivatives were all less efficient inhibitors of the tested 2OG oxygenases than 2,4-PDCA itself, except for the C5 F-substituted 2,4-PDCA derivative which inhibited AspH with a similar efficiency as 2,4-PDCA. Notably, the introduction of a F- or CF_3_-substituent at the C5 position of 2,4-PDCA results in a substantial increase in selectivity for AspH over KDM4E compared to 2,4-PDCA. Crystallographic studies inform on the structural basis of our observations, which exemplifies how a small change on a 2OG analogue can make a substantial difference in the potency of 2OG oxygenase inhibition.

## Highlights

• Synthesis of fluorinated pyridine-2,4-dicarboxylic acid (2,4-PDCA) derivatives
• Fluorinated 2,4-PDCA derivatives inhibit 2-oxoglutarate dependent oxygenases
• 2,4-PDCA C5 substituents increase the selectivity for AspH over KDM4 inhibition

## Graphical abstract

**Figure:**
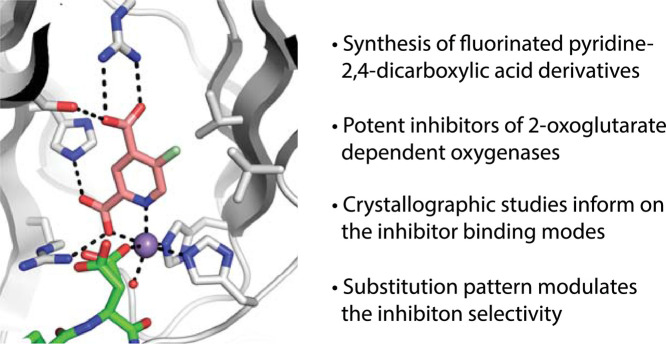

## 1 Introduction

**Fig. 1:**
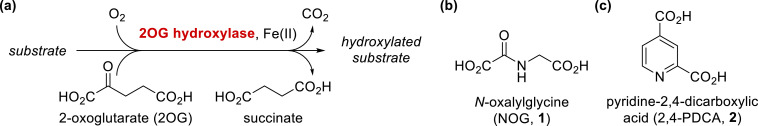
2OG hydroxylase catalysis and widely-used broad-spectrum inhibitors. a) 2OG hydroxylases couple substrate oxidation to the oxidative decarboxylation of 2OG using Fe(II) as a cofactor; b and c) the broad-spectrum 2OG oxygenase inhibitors: b) *N*-oxalylglycine (NOG, **1**) and c) pyridine-2,4-dicarboxylate (2,4-PDCA, **2**).

2-Oxoglutarate (2OG)-dependent oxygenases couple two electron substrate oxidations with the oxidative decarboxylation of 2OG to give succinate and CO_2_; they employ Fe(II) as a cofactor [1]. 2OG dependent hydroxylases have validated functions in human biology (Fig. 1a); for example, they act as sensors in the hypoxic response, *i.e.* the hypoxia inducible transcription factor-1α (HIF-1α) prolyl hydroxylases PHD1–3 together with the asparaginyl hydroxylase factor inhibiting the hypoxia inducible transcription factor-1α (FIH) catalyze the hydroxylation of HIF-α substrates in an O_2_-dependent manner [2]. Inhibiting human PHDs is of demonstrated therapeutic relevance for the treatment of anemia in patients with dialysis-dependent chronic kidney disease [3]. The development of potent and selective inhibitors for 2OG hydroxylases other than the PHDs is of basic scientific (for use in functional assignment studies) and therapeutic interest, in the latter case for diseases including cancer [4]. For example, aspartate/asparagine-β-hydroxylase (AspH), which catalyzes the stereoselective C3 hydroxylation of Asp/Asn-residues that are part of specific disulfide isomers of epidermal growth factor-like domains (EGFDs) [5], [6], [7], and certain JmjC lysine-specific *N*^ε^-demethylases, which catalyze the *N*^ε^-lysine demethylation of histones via initial *N*^ε^-methyl-group hydroxylation followed by fragmentation to give formaldehyde as a coproduct (Supporting Figure S1), are current medicinal chemistry targets for cancer treatment [8], [9], [10], [11], [12].

Broad-spectrum 2OG oxygenase inhibitors are described, including the natural product *N*-oxalylglycine (NOG, **1**; Fig. 1b) [13] and pyridine-2,4-dicarboxylate (2,4-PDCA, **2**; Fig. 1c) [4]. These broad-spectrum inhibitors show distinct selectivity profiles; for example, 2,4-PDCA efficiently inhibits AspH and some JmjC KDMs [14, 15]. By contrast, 2,4-PDCA is only a weak inhibitor of the PHDs and of FIH [15], which catalyzes the stereoselective C3 hydroxylation of Asn/Asp/His/Ser/Leu-residues (Supporting Figure S1) [16, 17]. For other classes of human 2OG oxygenases, such as the ribosomal oxygenase 2 (RIOX2; MYC-induced nuclear antigen 53, MINA53), which catalyzes the stereoselective C3 hydroxylation of His39 of the 60S ribosomal protein L27a (RPL27A) [18], the potency of these broad-spectrum inhibitors has not been reported.

Introducing substituents on the scaffold of broad-spectrum 2OG oxygenase inhibitors is a viable strategy to enhance inhibitor selectivity. For example, substituting the glycine of NOG by D-phenylalanine led to the development of the (partially) selective FIH inhibitor *N*-oxalyl-D-phenylalanine (NOFD) [19]. Analogous strategies for the identification of more selective 2,4-PDCA-based 2OG oxygenase inhibitors by introducing substituents at the C3 position of the pyridine ring have been largely unsuccessful in improving the selectivity pattern of 2,4-PDCA [15, 20]. We anticipated that the introduction of F- and CF_3_-substituents at the C3 and C5 position of the 2,4-PDCA scaffold might affect inhibitor selectivity due to the particular electronic and pharmacokinetic properties of fluorinated molecules, as being increasingly exploited in medicinal chemistry [21, 22]. Here we present proof-of-concept studies on how the introduction of substituents at the C5 position of 2,4-PDCA, which has not previously been investigated in structure activity relationship studies on 2OG oxygenases, increases the selectivity of 2,4-PDCA for AspH.

## 2 Results and discussion

### 2.1 Synthesis of F- and CF_3_-substituted 2,4-PDCA derivatives

**Scheme 1:**
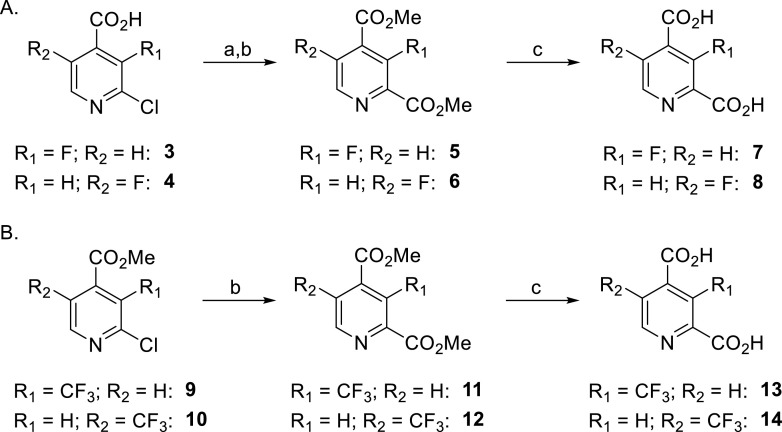
Synthesis of F- and CF_3_-substituted 2,4-PDCA derivatives. Reagents and conditions: a) SOCl_2_, MeOH, reflux, 59–93%; b) CO (1.5 atm), Cl_2_Pd-*rac*-BINAP (1 mol%), Hünig's base, MeOH, 100 °C (sand bath temperature, sealed flask), 89–94%; c) LiOH, MeOH/H_2_O, 0 °C to rt, 86–99%.

2,4-PDCA derivatives bearing F- or CF_3_-substituents at the C3 or C5 position were synthesized from commercially sourced F- or CF_3_-substituted isonicotinic acid derivatives in three or two steps, respectively, employing an analogous strategy to that used for the synthesis of C3 aminoalkyl-substituted 2,4-PDCA derivatives [15]. For example, 2-chloro-3-fluoroisonicotinic acid (**3**) was converted into its methyl ester, which was then submitted to Pd-catalyzed carbonylation [23] to afford dimethyl 3-fluoropyridine-2,4-dicarboxylate (**5**) (77%, over two steps; Scheme 1A). The purified 3-fluoropyridine-2,4-dicarboxylate (**7**), which is suitable for performing *in vitro* biochemical and biophysical studies as 2OG oxygenase inhibition, was obtained in 86% yield from dimethyl ester **5** after lithium hydroxide-mediated saponification and removal of excess base by acidic ion exchange chromatography. In a similar manner, 5-fluoropyridine-2,4-dicarboxylate (**8**) was obtained from 2-chloro-5-fluoroisonicotinic acid (**4**) (82%, over three steps; Scheme 1A). The corresponding purified 3- and 5-trifluoromethylated pyridine-2,4-dicarboxylates **13** and **14** were obtained in 79% and 89% yields, respectively, over two steps from the appropriate methyl trifluoromethylisonicotinic acid esters **9** and **10** (Scheme 1B).

### 2.2 F- and CF_3_-substituted 2,4-PDCA derivatives inhibit human 2OG oxygenases

The ability of the synthetic F- or CF_3_-substituted 2,4-PDCA derivatives **7, 8, 13**, and **14** to inhibit human 2OG oxygenases was assessed with selected isolated recombinant enzymes (Table 1). 2,4-PDCA (**2**) was included in the assays to enable comparison of the effects of the F- or CF_3_-substituents on the potency. Three representative human 2OG oxygenases, for which structures in complex with 2,4-PDCA have been reported, were employed in the inhibition studies: AspH [14], FIH [24], and KDM4E [25]. KDM4E was used as a representative JmjC KDM; it catalyzes the demethylation of histone 3 (H3) *N*^ε^-di- and trimethylated Lys9 (H3K9me3/me2) [10], [11], [12]. The inhibitory effect of 2,4-PDCA and its derivatives was also investigated for human RIOX2, though a structure of RIOX2 in complex with 2,4-PDCA has not yet been reported. A structure of the phylogenetically related ribosomal 2OG oxygenase RIOX1 (also known as nucleolar protein 66, NO66) with 2,4-PDCA (PDB ID: 4DIQ) suggests that 2,4-PDCA inhibits this subclass of 2OG oxygenases [26, 27]. Half maximum inhibitory concentrations (IC_50_-values) of 2,4-PDCA and its four derivatives were determined using solid phase extraction coupled to mass spectrometry (SPE-MS) inhibition assays, which directly monitor peptide hydroxylation (+16 Da; for AspH [14, 28], FIH [29], RIOX2 [30]) or demethylation (-14 and -28 Da; for KDM4E [31]) (Supporting Figure S1).

**Table 1:** F- and CF_3_-substituted 2,4-PDCA derivatives inhibit human 2OG oxygenases.

					
^a,b)^**AspH**	0.03 ± 0.01	0.11 ± 0.01	0.05 ± 0.01	>50	4.2 ± 1.0
^a,c)^**FIH**	4.7 ± 1.6	>50	>50	^d)^inactive	^d)^inactive
^a,e)^**KDM4E**	0.29 ± 0.05	1.3 ± 0.2	1.6 ± 0.3	^d)^inactive	^d)^inactive
^a,f)^**RIOX2**	4.0 ± 1.1	>50	>50	^d)^inactive	^d)^inactive

a) Mean of two independent runs (n = 2; mean ± SD); b) using 0.05 μM His_6_-AspH_315–758_ and 1.0 μM of a thioether-linked cyclic peptide based on human coagulation factor X (hFX, amino acids 101–119; hFX-CP_101–119_) [14, 28]; c) using 0.15 μM His_6_-FIH and 5.0 μM HIF-1α C-terminal transactivation domain fragment (HIF-1α CAD, amino acids 788–822) [29]; d) 2,4-PDCA derivatives were termed inactive if 2OG oxygenase inhibition was not observed in the tested concentration range (100 – 0.002 μM) as shown in Supporting Figure S3; e) using 0.15 μM KDM4E and 10.0 μM of a variant of a histone 3 fragment (H3_1–15_K9me3, amino acids 1–15) [31]; f) using 0.15 μM His_6_-RIOX2_26–465_ and 5.0 μM of RPL27A_31–49_ peptide (Supplementary Information Section 5) [30].

The SPE-MS inhibition assays were of high quality as revealed by Z′-factors > 0.5 (Supporting Figure S2) [32]. The Hill slopes [33] of the inhibition curves of 2,4-PDCA and active derivatives were near the theoretical value of -1 (Supporting Figure S3), as predicted for single molecules competing with 2OG for binding to the active site. The IC_50_-values of 2,4-PDCA (**2**) obtained for AspH, FIH, and KDM4E are in the ranges of those reported (Table 1) [15]. The inhibition of AspH by 2,4-PDCA is about an order of magnitude more efficient than of KDM4E as judged by IC_50_-values (*i.e.* IC_50_ ~ 0.03 μM for AspH and IC_50_ ~ 0.29 μM for KDM4E; Table 1), in part reflecting the different assay conditions used (final assay concentrations: KDM4E: 0.15 μM; AspH: 0.05 μM; Table 1). As anticipated based on a crystal structure of RIOX1 in complex with 2,4-PDCA (PDB ID: 4DIQ), efficient inhibition of RIOX2 by 2,4-PDCA was observed (IC_50_ ~ 4.0 μM, Table 1). 2,4-PDCA inhibits RIOX2 with similar potency as FIH (IC_50_ ~ 4.7 μM, Table 1), *i.e.* more than two orders of magnitude less efficiently than it inhibits AspH. This information should be of use in the screening and design of improved RIOX2 inhibitors; RIOX2 is a proposed anti-cancer medicinal chemistry target [34].

The F- and CF_3_-substituted 2,4-PDCA derivatives **7, 8, 13**, and **14** are all less efficient 2OG oxygenase inhibitors than 2,4-PDCA (Table 1), with the exception of the C5 F-substituted 2,4-PDCA derivative **8** which inhibits AspH with a similar potency as 2,4-PDCA (IC_50_ ~ 0.05 and 0.03 μM, respectively). The reduced inhibition observed for the F- and CF_3_-substituted 2,4-PDCA derivatives may reflect (in part) their weakened coordination to Fe(II) due to their reduced electron donating capacity caused by the electron withdrawing F- or CF_3_-substituents. In general, the 2,4-PDCA derivatives bearing CF_3_-substituents were less efficient 2OG oxygenase inhibitors than those bearing F-substituents (Table 1). Considering that the electron withdrawing effect of the F- and CF_3_-substituents on the central pyridine heterocyclic ring are likely in the same range (the CF_3_ group electronegativity is reported to be in the range of chlorine substituents [35]), the reduced 2OG oxygenase inhibition of 2,4-PDCA derivatives bearing CF_3_-substituents might relate to the steric repulsion of the CF_3_-substituent with side chains of active site residues; note, the A-value of a CF_3_-group is between those of isopropyl- and *tert*-butyl-groups [36, 37]. Alternatively, it may relate to the interaction of the CF_3_-substituent with the adjacent C4 (and C2 for **13**) 2,4-PDCA derivative carboxylate groups, potentially limiting their ability to engage in interactions with the side chains of Arg735, His690, Arg688, and Ser668 and the active site metal ion due to limited rotational freedom. No activity of the C3 or C5 CF_3_-substituted 2,4-PDCA derivatives **13** and **14** was observed against KDM4E, FIH, and RIOX2 (Table 1 and Supporting Figure S3), demonstrating how a relatively small modification to the broad-spectrum inhibitor 2,4-PDCA can cause a large difference in potency.

The IC_50_-values for both the C3 and C5 F-substituted 2,4-PDCA derivatives increase in the order of AspH < KDM4E < FIH ~ RIOX2 (Table 1). Substantial inhibition was only observed for AspH and KDM4E, while incomplete inhibition curves or no inhibition was observed for FIH and RIOX2 (Supporting Figure S3). Notably, the inhibition of AspH by 2,4-PDCA derivatives bearing a CF_3_- and, to a lesser extent, a F-substituent at the C5 position appears to be more efficient than by those bearing a CF_3_- or F-substituent at the C3 position. Consequently, the C5 F-substituted 2,4-PDCA derivative **8** shows a threefold increase in selectivity for AspH over KDM4E with respect to 2,4-PDCA and the 2,4-PDCA derivative **7**, and the C5 F-substituted 2,4-PDCA derivative **14** was only active against AspH, though much less potent than the C3 or C5 F-substituted 2,4-PDCA derivatives (Table 1).

### 2.3 Crystallography

To investigate the molecular reasons that determine why the inhibition of AspH by 2,4-PDCA derivatives bearing a CF_3_- or F-substituent at the C5 position appears to be more efficient than by those bearing a CF_3_- or F-substituent at the C3 position, crystallization studies with AspH and the fluorinated 2,4-PDCA derivatives were initiated. AspH was successfully crystallized in the presence of the F-substituted 2,4-PDCA derivatives **7** or **8**, Mn(II) substituting for Fe(II), and the synthetic hFX-EGFD1_86–124_–4Ser substrate peptide [6] (Supporting Figure S4), which mimics the EGFD1 of the reported AspH substrate human coagulation factor X (hFX) [38, 39], whereas crystallization in the presence of the CF_3_-substituted 2,4-PDCA derivatives **13** and **14** was unsuccessful. The structures were solved by molecular replacement using a reported AspH structure (PDB ID: 5JTC) [14] as a search model. In the presence of **7**, AspH crystallized in the *P*2_1_2_1_2_1_ space group (1.66 Å resolution, a single AspH molecule is in the asymmetric unit; Supporting Figure S5), in agreement with reported AspH structures [6, 14, 40]. By contrast, in the presence of **8**, AspH crystallized in the *P*1 space group with two molecules in the asymmetric unit, both of which were observed to bind to the inhibitor, but only one of which was bound to the substrate (1.75 Å resolution; Supporting Figure S6).

**Fig. 2:**
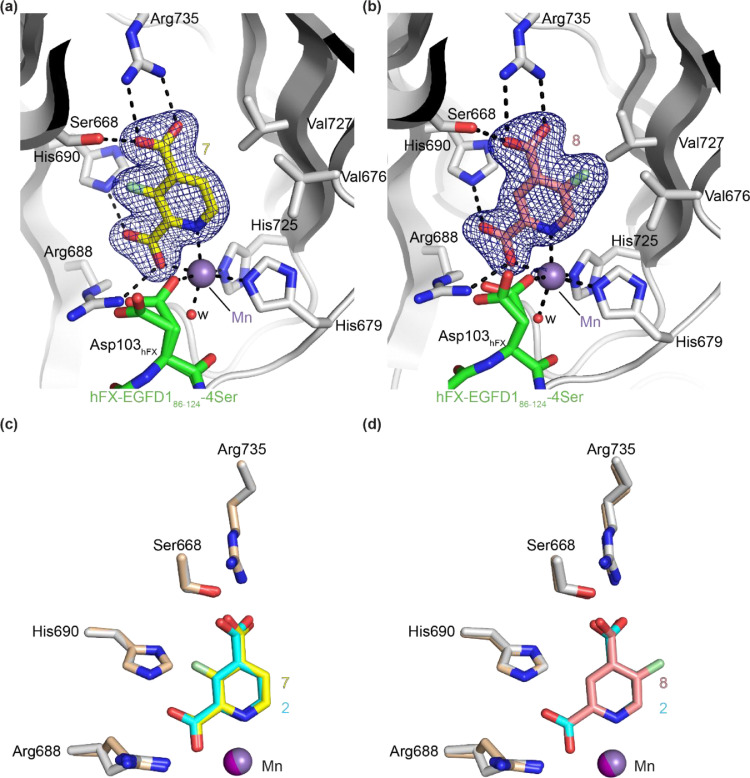
Binding mode of C3 and C5 F-substituted 2,4-PDCA derivatives with AspH. Colors: gray: His_6_-AspH_315–758_; yellow: carbon-backbone of 3-fluoropyridine-2,4-dicarboxylate (**7**); salmon: carbon-backbone of 5-fluoropyridine-2,4-dicarboxylate (**8**); lavender blue: Mn; green: carbon-backbone of the hFX-EGFD1_86–124_–4Ser peptide (Supporting Figure S4); red: oxygen; blue: nitrogen; pale green: fluorine. w: water. a and b) Representative OMIT electron density map (mF_o_-DF_c_) contoured to (a) 7.0σ around **7** of the AspH:**7** structure and (b) 7.0σ around **8** of the AspH:**8** structure, respectively; c and d) superimposition of a view of the AspH:**2** structure (pale brown: His_6_-AspH_315–758_; pink: Mn; cyan: carbon-backbone of 2,4-PDCA **2**) with (c) the AspH:**7** structure and (d) the AspH:**8** structure, respectively.

Clear electron density for both **7** and **8** was observed in the AspH active site (Fig. 2a and b), in agreement with the 2OG competitive inhibition mechanism observed for 2,4-PDCA [14, 28]. Substituting 2,4-PDCA by the 2,4-PDCA derivative **7** or **8** in the AspH active site neither changes the overall protein fold nor the hFX-EGFD1_86–124_–4Ser peptide conformation (Supporting Figures S7-S9). The F-substituted 2,4-PDCA derivatives bind to the AspH active site in a manner similar to 2,4-PDCA (Fig. 2c and d), *i.e.* the C2 carboxylates are positioned to interact with Arg688 (**7**: 2.8 Å; **8**: 2.7 Å) and His690 (**7**: 2.9 Å; **8**: 2.8 Å) and bind the metal ion (**7**: 2.2 Å; **8**: 2.2 Å) together with the pyridine nitrogen atoms (**7**: 2.3 Å; **8**: 2.3 Å); the C4 carboxylates are positioned to interact with Ser668 (**7**: 2.7 Å; **8**: 2.6 Å) and Arg735 (**7**: 2.6 and 2.9 Å; **8**: 2.6 and 3.1 Å).

The two AspH structures inform on the molecular rationale for the observed differences in the inhibitory potency of the C3 and C5 F-substituted 2,4-PDCA derivatives **7** and **8** and, by analogy, the C3 and C5 CF_3_-substituted 2,4-PDCA derivatives **13** and **14** (Table 1). The F-substituent at the C3 position of **7** is in close proximity to the side chains of Ile737, Ile739, Ser668, and His690; bulkier substituents at the C3 position of 2,4-PDCA (such as the CF_3_-group in **13**) likely clash with these side chains hampering efficient binding of the molecules to the AspH active site (*i.e.* IC_50_ > 50 μM for **13**, Table 1). By contrast, the F-substituent at the C5 position of **8** faces towards the hydrophobic side chains of Val676 and Val727 which, together with those of Trp625 and Met670, form a more spacious hydrophobic pocket (compared to that available for C3 substituents), which can also accommodate bulkier substituents than F, *e.g.* the C5 CF_3_-substituent of **14** (IC_50_ ~ 4.2 μM, Table 1) [40]. The F-substituent of **8** can potentially engage in hydrophobic interactions with the proximal hydrophobic pocket of AspH, which could rationalize the observed similar potency of **8** and 2,4-PDCA in inhibiting AspH (IC_50_ ~ 0.05 and 0.03 μM, respectively).

We anticipate that the structure activity relationship results presented here will help to enable the development of 2OG competitive AspH inhibitors for use in validating AspH as a target for cancer therapy and diagnostics; AspH is reported to be upregulated on the surface of cancer cells potentially obviating the necessity for the 2,4-PDCA derivative to penetrate the cell wall [41, 42]. By contrast, most 2OG oxygenases are intracellular, including the RIOXs and JmjC KDMs, which are potential cancer targets [[10], [11], [12], 30]. 2,4-PDCA dimethylesters have been used in cell-based and *in vivo* inhibition studies of 2OG oxygenases [43, 44], the presence of hydrophobic F- or CF_3_-substituents on the scaffold of 2,4-PDCA might further increase the cell-wall permeability of the 2,4-PDCA derivatives.

Other potential additional applications of the F-substituted 2,4-PDCA derivatives include, for example, their use as electron deficient substrates for nucleophilic aromatic substitution reactions to label active site cysteine residues, which are present in the human DNA-modifying and 2OG-dependent ten-eleven translocation (TET) enzymes [45]. The F-substituted 2,4-PDCA derivatives may also be of use as ^19^F NMR probes in fluorine chemical shift anisotropy and exchange high-throughput screening for protein binding [46, 47]. Recent advances in fluorination reactions suitable for the rapid late-stage introduction of radioactive ^18^F atoms into hetereoaromatic scaffolds [48, 49], including in substituted pyridines such as fluorinated derivatives of the multiple sclerosis drug 4-aminopyridine [50], [51], [52], suggest the F-substituted 2,4-PDCA derivatives might be useful scaffolds for positron emission tomography (PET) studies.

## 3 Conclusions

F- or CF_3_-substituted derivatives of the broad-spectrum 2OG oxygenase inhibitor 2,4-PDCA were synthesized and their inhibitory activity determined for a set of human 2OG hydroxylases, which were already known to bind 2,4-PDCA [14, 24, 25]. Both the C3 and C5 F-substituted 2,4-PDCA derivatives were efficient AspH and KDM4E inhibitors, displaying a similar potency as 2,4-PDCA for AspH inhibition and an about fourfold reduced potency compared to 2,4-PDCA for KDM4E inhibition. Unlike 2,4-PDCA, the F- or CF_3_-substituted 2,4-PDCA derivatives investigated did not inhibit FIH or RIOX2. The selectivity profile of the F- and CF_3_-substituted 2,4-PDCA derivatives is similar to that of 2,4-PDCA, with the exceptions of 2,4-PDCA derivatives **8** and **14** for which a substantial increase in selectivity for AspH over KDM4E was observed, whilst in the case of **8** (but not **14**) maintaining potent AspH inhibition; an unprecedented observation with respect to 2OG oxygenase inhibition by 2,4-PDCA derivatives [15, 20]. Crystallographic analyses provide a rationale for the observed selectivity increase and thus will help to enable the design of more selective AspH inhibitors suitable for *in vivo* use.

## 4 Experimental

### 4.1 General information

Unless otherwise stated, all reagents were from commercial sources (Sigma-Aldrich, Inc.; Fluorochem Ltd) and used as received. Anhydrous solvents were from Sigma-Aldrich, Inc. and kept under an atmosphere of nitrogen. Solvents, liquids, and solutions were transferred using nitrogen-flushed stainless steel needles and syringes. Milli-Q® Ultrapure (MQ-grade) water was used for buffers; LCMS grade solvents (Merck) were used for solid phase extraction coupled to mass spectrometry (SPE-MS).

Purifications were performed using an automated Biotage Isolera One purification machine (wavelength monitored: 254 and 280 nm) equipped with pre-packed Biotage® SNAP KP-Sil or Biotage® SNAP Ultra flash chromatography cartridges. The cartridge size and solvent gradients (in column volumes, CV) used, are specified in the individual experimental procedures. HPLC grade solvents (ethyl acetate and cyclohexane; Sigma-Aldrich Inc.) were used for reaction work-ups, extractions, and purifications.

Thin layer chromatography (TLC) was carried out using Merck silica gel 60 F_254_ TLC plates and visualized under UV light. Melting points (m.p.) were determined using a Stuart SMP-40 automated melting point apparatus. Infrared (IR) spectroscopy was performed using a Bruker Tensor-27 Fourier transform infrared (FT-IR) spectrometer. High-resolution mass spectrometry (HRMS) was performed using electro-spray ionization (ESI) mass spectrometry (MS) in the positive or negative ionization modes employing a Thermo Scientific Exactive mass spectrometer (ThermoFisher Scientific); data are presented as a mass-to-charge ratio (*m/z*).

Nuclear magnetic resonance (NMR) spectroscopy was performed using a Bruker AVANCE AVIIIHD 600 machine equipped with a 5 mm BB-F/1H Prodigy N_2_ cryoprobe. Chemical shifts for protons are reported in parts per million (ppm) downfield from tetramethylsilane and are referenced to residual protium in the NMR solvent (CDCl_3_: *δ* = 7.28 ppm; D_2_O: *δ* = 4.79 ppm). For ^13^C NMR, chemical shifts are reported in the scale relative to the NMR solvent (*i.e.* CDCl_3_: *δ* = 77.00 ppm). For ^19^F NMR, chemical shifts are reported in the scale relative to CFCl_3_. NMR data are reported as follows: chemical shift, multiplicity (s: singlet, d: doublet, dd: doublet of doublets, t: triplet, q: quartet, m: multiplet), coupling constant (*J*, Hz; accurate to 0.1 Hz), and integration. ^13^C NMR chemical shift numbers in brackets indicate close signals that can be differentiated taking into account second decimal numbers.

### 4.2 General procedures

#### 4.2.1 General procedure A

Thionyl chloride (1.5 equiv.) was added dropwise to a solution of 3- or 5-fluoro-2-chloroisonicotinic acid (1.0 equiv.) in anhydrous methanol (0.6 M) at ambient temperature under a nitrogen atmosphere. The reaction mixture was stirred under reflux for 2 h, then cooled to ambient temperature and concentrated. The residue was dissolved in ethyl acetate, washed twice with saturated aqueous NaHCO_3_ solution, then once with brine. The organic solution was dried over anhydrous Na_2_SO_4_, filtered, evaporated, and purified by column chromatography to afford the corresponding methyl esters which were used in the next reaction following General Procedure B.

#### 4.2.2 General procedure B

*N,N*-Diisopropylethylamine (Hünig's base; 1.5 equiv.) was added to a solution of a C3 or C5 fluoro/trifluoromethyl-substituted methyl 2-chloroisonicotinate (1.0 equiv.) and dichloro[2,2′-bis(diphenylphosphino)-1,1′-binaphthyl]palladium(II) [(*rac*-BINAP)PdCl_2_] (0.01 equiv.) in anhydrous methanol (0.2 M) in a 250 mL J-Young Schlenk tube under an ambient temperature. Carbon monoxide gas (synthesis grade) was bubbled through the solution for 10 minutes. *Caution*: Carbon monoxide is a highly toxic and flammable gas; it should be handled in a well-vented fume cupboard taking appropriate safety measures. The Schlenk tube was then sealed under CO-pressure (~1.5−2.0 atm) and placed in a sand bath; the tube was then heated with stirring behind a safety shield at 100 °C for 18−20 h. The reaction mixture was cooled to ambient temperature, then concentrated and purified by column chromatography to afford the corresponding C3 or C5 fluoro/trifluoromethyl-substituted dimethyl pyridine-2,4-dicarboxylates which were used in the next reaction following General Procedure C.

#### 4.2.3 General procedure C

An aqueous solution of lithium hydroxide (0.4 M, 2.8 equiv.) was added to a solution of C3 or C5 fluoro/trifluoromethyl-substituted dimethyl pyridine-2,4-dicarboxylate (1.0 equiv.) in methanol (0.2 M, HPLC grade) under an ambient atmosphere at 0 °C. The reaction mixture was allowed to slowly warm to ambient temperature overnight (14 – 18 h). The methanol was then removed under reduced pressure. The aqueous reaction mixture was extracted three times with dichloromethane (the organic extracts were discarded) and the aqueous phase was acidified (pH ≈ 7.0 to 7.7) using Dowex® 50XW8 (H^+^-form, mesh 200–400). The mixture was filtered and lyophilized to afford the solid C3 or C5 fluoro/trifluoromethyl-substituted pyridine-2,4-dicarboxylate. The crude product was sufficiently pure as judged by ^1^H and ^13^C NMR and used without further purification in the biological assays. pKa-values for the 2,4-PDCA derivatives were not determined, thus, some might have actually been isolated as the corresponding mono- or dilithium salts.

### 4.3 Synthetic procedures and analytical data

#### 4.3.1 Methyl 2-chloro-3-fluoroisonicotinate (**15**)

According to General Procedure A, methyl ester **15** (0.68 g, 59%) was obtained from commercially sourced 2-chloro-3-fluoroisonicotinic acid **3** (1.06 g, 6.0 mmol), following column chromatography (10 g Ultra cartridge; 35 mL/min; initially, 100% cyclohexane (2 column volumes, CV), followed by a linear gradient (15 CV): 0%→20% ethyl acetate in cyclohexane). White solid, m.p.: 43−45 °C; ^1^H NMR (600 MHz, 300 K, CDCl_3_): *δ* = 8.31 (d, *J* = 4.9 Hz, 1H), 7.71 (t, *J* = 4.8 Hz, 1H), 3.99 ppm (s, 3H); ^19^F NMR (565 MHz, 300 K, CDCl_3_): *δ* = −116.2 ppm (d, *J* = 4.1 Hz, 1F); ^13^C NMR (150 MHz, 300 K, CDCl_3_): *δ* = 162.4 (d, *J* = 3.3 Hz), 152.8 (d, *J* = 274.1 Hz), 144.5 (d, *J* = 7.7 Hz), 141.2 (d, *J* = 20.7 Hz), 127.1 (d, *J* = 9.6 Hz), 123.9, 53.2 ppm (d, *J* = 2.2 Hz); IR (film): ṽ = 3109, 3022, 2966, 1732, 1458, 1429, 1397, 1272, 1257, 1204, 1136, 1086, 977 cm^–1^; HRMS (ESI): *m/z* calculated for C_7_H_6_O_2_NClF [M+H]^+^: 190.0066, found: 190.068.

#### 4.3.2 Dimethyl 3-fluoropyridine-2,4-dicarboxylate (**5**)

According to General Procedure B, dimethyl ester **5** (0.72 g, 94%) was obtained from methyl 2-chloro-3-fluoroisonicotinate (**15**) (0.68 g, 3.6 mmol), following column chromatography (25 g KP-Sil cartridge; 50 mL/min; initially, 100% cyclohexane (3 CV), followed by a linear gradient (10 CV): 0%→50% ethyl acetate in cyclohexane). Pale yellow solid, m.p.: 57−59 °C; ^1^H NMR (600 MHz, 300 K, CDCl_3_): *δ* = 8.65 (d, *J* = 4.7 Hz, 1H), 7.96 (t, *J* = 4.8 Hz, 1H), 4.05 (s, 3H), 4.02 ppm (s, 3H); ^19^F NMR (565 MHz, 300 K, CDCl_3_): *δ* = −118.3 ppm (d, J = 5.9 Hz, 1F); ^13^C NMR (150 MHz, 300 K, CDCl_3_): *δ* = 162.9 (d, *J* = 6.0 Hz), 162.7 (d, *J* = 2.1 Hz), 157.0 (d, *J* = 283.0 Hz), 145.3 (d, *J* = 7.3 Hz), 138.9 (d, *J* = 11.0 Hz), 128.3, 127.9 (d, *J* = 10.1 Hz), 53.3, 53.2 ppm; IR (film): ṽ = 3009, 2958, 1736, 1466, 1437, 1418, 1324, 1283, 1238, 1205, 1177, 1136, 1086, 993, 956 cm^–1^; HRMS (ESI): *m/z* calculated for C_9_H_9_O_4_NF [M+H]^+^: 214.0510, found: 214.0511.

#### 4.3.3 3-Fluoropyridine-2,4-dicarboxylic acid (**7**)

Dicarboxylic acid **7** (160 mg, 86%) was obtained from dimethyl 3-fluoropyridine-2,4-dicarboxylate **5** (213 mg, 1.0 mmol) according to General Procedure C. White solid, m.p.: >230 °C (decomposition); ^1^H NMR (600 MHz, 300 K, D_2_O): *δ* = 8.42 (d, *J* = 4.9 Hz, 1H), 7.67 ppm (t, *J* = 5.0 Hz, 1H); ^19^F NMR (565 MHz, 300 K, D_2_O): *δ* = −128.1 ppm (d, *J* = 4.1 Hz, 1F); ^13^C NMR (150 MHz, 300 K, D_2_O): *δ* = 169.8, 169.2 (d, *J* = 3.5 Hz), 153.1 (d, *J* = 259.9 Hz), 143.8 (d, *J* = 5.7 Hz), 143.1 (d, *J* = 19.0 Hz), 137.1 (d, *J* = 16.2 Hz), 124.8 ppm (d, *J* = 5.4 Hz); IR (film): ṽ = 3352, 1596, 1551, 1462, 1372, 1241, 1226, 1181, 1089 cm^–1^; HRMS (ESI): *m/z* calculated for C_7_H_3_O_4_NF [M−H]^−^: 184.0052, found: 184.0047.

#### 4.3.4 Methyl 2-chloro-5-fluoroisonicotinate (**16**)

According to General Procedure A, methyl ester **16** (2.67 g, 93%) was obtained from commercially sourced 2-chloro-5-fluoroisonicotinic acid **4** (2.67 g, 15.2 mmol), following column chromatography (50 g KP-Sil cartridge; 50 mL/min; initially, 100% cyclohexane (2 CV), followed by a linear gradient (8 CV): 0%→20% ethyl acetate in cyclohexane). White solid, m.p.: 49−51 °C; ^1^H NMR (600 MHz, 300 K, CDCl_3_): *δ* = 8.41 (s, 1H), 7.81 (d, *J* = 5.0 Hz, 1H), 4.00 ppm (s, 3H); ^19^F NMR (565 MHz, 300 K, CDCl_3_): *δ* = −128.4 ppm (d, *J* = 4.8 Hz, 1F); ^13^C NMR (150 MHz, 300 K, CDCl_3_): *δ* = 162.1 (d, *J* = 3.4 Hz), 156.3 (d, *J* = 268.4 Hz), 146.7 (d, *J* = 4.1 Hz), 140.0 (d, *J* = 28.0 Hz), 128.2 (d, *J* = 10.5 Hz), 125.4, 53.3 ppm (d, *J* = 1.6 Hz); IR (film): ṽ = 3098, 3031, 2961, 1721, 1462, 1438, 1357, 1269, 1246, 1212, 1132, 1083, 961 cm^–1^; HRMS (ESI): *m/z* calculated for C_7_H_6_O_2_NClF [M+H]^+^: 190.0066, found: 190.0068.

#### 4.3.5 Dimethyl 5-fluoropyridine-2,4-dicarboxylate (**6**)

According to General Procedure B, dimethyl ester **6** (1.64 g, 89%) was obtained from methyl 2-chloro-5-fluoroisonicotinate (**16**) (1.9 g, 10.0 mmol), following column chromatography (50 g KP-Sil cartridge; 50 mL/min; initially, 100% cyclohexane (2 CV), followed by a linear gradient (12 CV): 0%→100% ethyl acetate in cyclohexane). White solid, m.p.: 87−89 °C; ^1^H NMR (600 MHz, 300 K, CDCl_3_): *δ* = 8.72 (d, *J* = 1.7 Hz, 1H), 8.63 (d, *J* = 5.8 Hz, 1H), 4.04 (s, 3H), 4.02 ppm (s, 3H); ^19^F NMR (565 MHz, 300 K, CDCl_3_): *δ* = −118.4 ppm (d, *J* = 5.6 Hz, 1F); ^13^C NMR (150 MHz, 300 K, CDCl_3_): *δ* = 164.0, 162.4 (d, *J* = 3.1 Hz), 158.8 (d, *J* = 276.2 Hz), 144.5 (d, *J* = 5.4 Hz), 140.8 (d, *J* = 26.5 Hz), 127.0, 126.1 (d, *J* = 8.8 Hz), 53.2 (d, *J* = 2.2 Hz), 53.1(9) ppm (d, *J* = 2.2 Hz); IR (film): ṽ = 3085, 3040, 3018, 2961, 1733, 1720, 1607, 1561, 1467, 1447, 1433, 1402, 1310, 1259, 1242, 1213, 1139, 1082, 991, 956 cm^–1^; HRMS (ESI): *m/z* calculated for C_9_H_9_O_4_NF [M+H]^+^: 214.0510, found: 214.0511.

#### 4.3.6 5-Fluoropyridine-2,4-dicarboxylic acid (**8**)

Dicarboxylic acid **8** (184 mg, 99%) was obtained from dimethyl 5-fluoropyridine-2,4-dicarboxylate **6** (213 mg, 1.0 mmol), along with some minor impurities which were not separated, according to General Procedure C. White solid, m.p.: >270 °C (decomposition); ^1^H NMR (600 MHz, 300 K, D_2_O): *δ* = 8.51 (d, *J* = 1.2 Hz, 1H), 8.06 ppm (d, *J* = 5.8 Hz, 1H); ^19^F NMR (565 MHz, 300 K, D_2_O): *δ* = −128.5 ppm (d, *J* = 5.6 Hz, 1F); ^13^C NMR (150 MHz, 300 K, D_2_O): *δ* = 171.7, 170.3, 156.7 (d, *J* = 259.8 Hz), 149.9 (d, *J* = 5.0 Hz), 138.1 (d, *J* = 27.2 Hz), 134.4 (d, *J* = 14.2 Hz), 123.6 ppm; IR (film): ṽ = 3245, 1603, 1436, 1380, 1296, 1221, 1096 cm^–1^; HRMS (ESI): *m/z* calculated for C_7_H_3_O_4_NF [M−H]^−^: 184.0052, found: 184.0047.

#### 4.3.7 Dimethyl 3-trifluoromethylpyridine-2,4-dicarboxylate (**11**)

According to General Procedure B, dimethyl ester **11** (0.99 g, 89%) was obtained from commercially sourced methyl 2-chloro-3-trifluoromethylisonicotinate (**9**) (1.0 g, 4.2 mmol), following column chromatography (25 g KP-Sil cartridge; 50 mL/min; initially, 100% cyclohexane (3 CV), followed by a linear gradient (20 CV): 0%→40% ethyl acetate in cyclohexane). Pale yellow solid, m.p.: 48−50 °C; ^1^H NMR (600 MHz, 300 K, CDCl_3_): *δ* = 8.90 (d, *J* = 4.9 Hz, 1H), 7.65 (d, *J* = 4.9 Hz, 1H), 4.03 (s, 3H), 4.00 ppm (s, 3H); ^19^F NMR (565 MHz, 300 K, CDCl_3_): *δ* = −56.8 ppm (s, 3F); ^13^C NMR (150 MHz, 300 K, CDCl_3_): *δ* = 165.5, 165.4, 152.5, 150.5 (q, *J* = 2.6 Hz), 140.9 (q, *J* = 2.4 Hz), 123.8, 122.1 (q, *J* = 274.7 Hz), 121.6 (q, *J* = 34.8 Hz), 53.6 (q, *J* = 8.4 Hz), 53.5 ppm (q, *J* = 8.3 Hz); IR (film): ṽ = 3015, 2960, 1743, 1588, 1560, 1437, 1415, 1320, 1297, 1272, 1214, 1187, 1148, 1109, 1034, 988, 955 cm^–1^; HRMS (ESI): *m/z* calculated for C_10_H_9_O_4_NF_3_ [M+H]^+^: 264.0478, found: 264.0478.

#### 4.3.8 3-Trifluoromethylpyridine-2,4-dicarboxylic acid (**13**)

Dicarboxylic acid **13** (210 mg, 89%) was obtained from dimethyl 3-trifluoromethylpyridine-2,4-dicarboxylate **11** (263 mg, 1.0 mmol) according to General Procedure C. White solid, m.p.: >220 °C (decomposition); ^1^H NMR (600 MHz, 300 K, D_2_O): *δ* = 8.68 (d, *J* = 5.1 Hz, 1H), 7.46 ppm (d, *J* = 5.1 Hz, 1H); ^19^F NMR (565 MHz, 300 K, D_2_O): *δ* = −57.4 ppm (s, 3F); ^13^C NMR (150 MHz, 300 K, D_2_O): *δ* = 173.5, 173.2, 155.5 (q, *J* = 2.9 Hz), 151.8, 148.3 (q, *J* = 2.7 Hz), 123.0 (q, *J* = 273.9 Hz), 120.6, 115.6 ppm (q, *J* = 32.8 Hz); IR (film): ṽ = 3358, 1591, 1555, 1462, 1375, 1289, 1239, 1136, 1117, 1039 cm^–1^; HRMS (ESI): *m/z* calculated for C_8_H_3_O_4_NF_3_ [M−H]^−^: 234.0020, found: 234.0020.

#### 4.3.9 Dimethyl 5-trifluoromethylpyridine-2,4-dicarboxylate (**12**)

According to General Procedure B, dimethyl ester **12** (1.03 g, 93%) was obtained from commercially sourced methyl 2-chloro-5-trifluoromethylisonicotinate (**10**) (1.0 g, 4.2 mmol), following column chromatography (25 g KP-Sil cartridge; 50 mL/min; initially, 100% cyclohexane (3 CV), followed by a linear gradient (15 CV): 0%→30% ethyl acetate in cyclohexane). Pale yellow solid, m.p.: 58−60 °C; ^1^H NMR (600 MHz, 300 K, CDCl_3_): *δ* = 9.13 (s, 1H), 8.45 (s, 1H), 4.08 (s, 3H), 4.02 ppm (s, 3H); ^19^F NMR (565 MHz, 300 K, CDCl_3_): *δ* = −59.8 ppm (s, 3F); ^13^C NMR (150 MHz, 300 K, CDCl_3_): *δ* = 164.4, 163.8, 151.6, 148.1 (q, *J* = 5.6 Hz), 140.0 (q, *J* = 2.1 Hz), 126.1 (q, *J* = 33.3 Hz), 124.5, 122.2 (q, *J* = 274.4 Hz), 53.6, 53.5 ppm; IR (film): ṽ = 3117, 3022, 2964, 1749, 1727, 1598, 1567, 1489, 1428, 1373, 1331, 1251, 1148, 1129, 1111, 1037, 982, 956 cm^–1^; HRMS (ESI): *m/z* calculated for C_10_H_9_O_4_NF_3_ [M+H]^+^: 264.0478, found: 264.0479.

#### 4.3.10 5-Trifluoromethylpyridine-2,4-dicarboxylic acid (**14**)

Dicarboxylic acid **14** (226 mg, 96%) was obtained from dimethyl 5-trifluoromethylpyridine-2,4-dicarboxylate **12** (263 mg, 1.0 mmol) according to General Procedure C. White solid, m.p.: >260 °C (decomposition); ^1^H NMR (600 MHz, 300 K, D_2_O): *δ* = 8.91 (s, 1H), 7.90 ppm (s, 1H); ^19^F NMR (565 MHz, 300 K, D_2_O): *δ* = −60.1 ppm (s, 3F); ^13^C NMR (150 MHz, 300 K, D_2_O): *δ* = 173.2, 171.4, 157.3, 147.7 (q, *J* = 2.4 Hz), 146.6 (q, *J* = 5.0 Hz), 123.2 (q, *J* = 273.0 Hz), 121.6 (q, *J* = 32.3 Hz), 120.3 ppm; IR (film): ṽ = 3427, 1601, 1557, 1441, 1390, 1361, 1321, 1279, 1199, 1158, 1138, 1045 cm^–1^.

### 4.4 2OG oxygenase inhibition assays

SPE-MS inhibition assays using human *N*-terminally His_6_-tagged AspH_315–758_ [14, 28], *N*-terminally His_6_-tagged FIH [29], and KDM4E [15, 31] were performed in independent duplicates as described in the cited literature; The standard deviation (SD) of two independent IC_50_ determinations (n = 2), each composed of technical duplicates, was calculated using GraphPad Prism 5. 2OG oxygenases were produced and purified as described in the cited literature, the peptide substrate for AspH was synthesized according to the cited literature while the peptide substrates for FIH and KDM4E were obtained from GL Biochem (Shanghai) Ltd (Shanghai, China). All peptide were prepared with C-terminal amides.

SPE-MS inhibition assays using recombinant human *N*-terminally His_6_-tagged RIOX2_26–465_ (human wild-type RIOX2 (amino acid sequence: 26–465) was produced in *E. coli* BL21 (DE3) cells using a pET-28a(+) vector and purified as reported [18, 26]) were performed as described below using the RPL27A_31–49_ substrate peptide (amino acids 31–49 of human RPL27A: GRGNAGGLHHHRINFDKYHP, H39 is the hydroxylation site of RIOX2 [18]; synthesized and purified by GL Biochem (Shanghai) Ltd from Shanghai, China). Details of the RIOX2 assay will be reported elsewhere [30].

Solutions of the 2OG derivatives (100% DMSO) were dry dispensed across 384-well polypropylene assay plates (Greiner) in a threefold and 11-point dilution series (100 μM top concentration) using an ECHO 550 acoustic dispenser (Labcyte). DMSO and 2,4-PDCA were used as negative and positive inhibition controls, respectively. The final DMSO concentration was kept constant at 0.5%_v/v_ throughout all experiments (using the DMSO backfill option of the acoustic dispenser). Each reaction was performed in technical duplicates in adjacent wells of the assay plates; additionally, assays were performed in two independent duplicates on different days using different DMSO inhibitor solutions. Cosubstrate/cofactor stock solutions (L-ascorbic acid, LAA: 50 mM in MQ-grade water; 2-oxoglutarate, 2OG: 10 mM in MQ-grade water; ammonium iron(II) sulfate hexahydrate, FAS, (NH_4_)_2_Fe(SO_4_)_2_•6H_2_O: 400 mM in 20 mM HCl diluted to 1 mM in MQ-grade water) were freshly prepared each day from commercially sourced solids (Sigma Aldrich).

The Enzyme Mixture (25 μL/well), containing His_6_-RIOX2_26–465_ (0.3 μM) in reaction buffer (50 mM HEPES, 50 mM NaCl, pH 7.5), was dispensed across the inhibitor-containing 384-well assay plates with a multidrop dispenser (ThermoFischer Scientific) at 20 °C under an ambient atmosphere. The plates were subsequently centrifuged (1000 rpm, 20 s) and incubated for 15 minutes at 20 °C. The Substrate Mixture (25 μL/well), containing RPL27A_31–49_ substrate peptide (10 μM), LAA (200 μM), 2OG (20 μM), and FAS (20 μM) in reaction buffer, was added using the multidrop dispenser. The plates were centrifuged (1000 rpm, 20 s) and, after incubating for 30 minutes, the enzyme reaction was stopped by the addition of 10%_v/v_ aqueous formic acid (5 μL/well). The plates were then centrifuged (1000 rpm, 30 s) and analysed by MS.

MS-analyses were performed using a RapidFire RF 365 high-throughput sampling robot (Agilent) attached to an iFunnel Agilent 6550 accurate mass quadrupole time-of-flight (Q-TOF) mass spectrometer operated in the positive ionization mode. Assay samples were aspirated under vacuum for 0.6 s and loaded onto a C4 solid phase extraction (SPE) cartridge. After loading, the C4 SPE cartridge was washed with 0.1%_v/v_ aqueous formic acid to remove non-volatile buffer salts (5.5 s, 1.5 mL/min). The peptide was eluted from the SPE cartridge with 0.1%_v/v_ formic acid in 80/20_v/v_ acetonitrile/water into the mass spectrometer (5.5 s, 1.4 mL/min) and the SPE cartridge re-equilibrated with 0.1%_v/v_ aqueous formic acid (0.5 s, 1.25 mL/min). The mass spectrometer parameters were: capillary voltage (4000 V), nozzle voltage (1000 V), fragmentor voltage (365 V), gas temperature (280 °C), gas flow (13 L/min), sheath gas temperature (350 °C), sheath gas flow (12 L/min), nebulizer pressure (40 psig). The *m/z* +4 charge states of the RPL27A_31–49_ substrate peptide and the hydroxylated product peptide were used to extract ion chromatogram data, peak areas were integrated using RapidFire Integrator software (Agilent). Data were exported into Microsoft Excel and used to calculate the % conversion of the hydroxylation reaction using the equation: % conversion = 100 x (integral hydroxylated product peptide) / (integral RPL27A_31–49_ substrate peptide + integral hydroxylated product peptide). Normalized dose-response curves (2,4-PDCA and DMSO controls) were obtained from the raw data by non-linear regression (GraphPad Prism 5) and used to determine IC_50_-values. The SD of two independent IC_50_ determinations (n = 2) was calculated using GraphPad Prism 5.

### 4.5 Crystallography and structure solutions

High-throughput crystallization experiments were performed in 96-well, 3-subwell, low profile Intelliplates (Art Robbins Instruments) using a Phoenix RE liquid dispensing robot (Art Robbins Instruments) and the PACT Premier crystallization screen (Molecular Dimensions). *N*-Terminally His_6_-tagged AspH_315–758_ (0.33 mM in 50 mM HEPES buffer, pH 7.5) was mixed with 1 mM MnCl_2_, 2 mM fluorinated 2,4-PDCA derivative, and the hFX-EGFD1_86–124_–4Ser peptide [6] (0.73 mM; Supporting Figure S4) as AspH substrate. Crystals were grown using the vapor diffusion method at 4 °C in 200 nL or 300 nL sitting drops with 2:1, 1:1 or 1:2 sample:well solution ratios; precipitants are listed in the Supporting Table S1. Crystals were cryo-protected using mother liquor supplemented with 20%_v/v_ glycerol before cryo-cooling in liquid N_2_. Data were collected at 100 K using synchrotron radiation at Diamond Light Source (DLS) beamlines I03 and I24. Data were indexed, integrated, and scaled using the Xia2 [53] strategy of the beamline auto-processing pipeline (Supporting Table S1).

The AspH crystal structures were determined by molecular replacement (MR) using the AutoMR (PHASER [54]) subroutine in PHENIX [55]. The search model used for MR was based on PDB ID 5JTC [14]. The structural model was improved by iterative cycles of manual re-building in COOT [56] and crystallographic refinement in phenix.refine [57] (refinement details are summarized in Supporting Table S1).

Crystal structure data for *N*-terminal His_6_-tagged AspH_315–758_ complexed to Mn, the fluorinated 2,4-PDCA derivatives (3-fluoropyridine-2,4-dicarboxylic acid, **7**; 5-fluoropyridine-2,4-dicarboxylic acid, **8**), and substrate peptide (hFX-EGFD1_86–124_–4Ser) are deposited in the protein data bank with PDB accession codes: 7MBI (AspH:**7**) and 7MBJ (AspH:**8**). PyMOL [58] was used for the generation of graphical representations; polder omit maps were calculated using Polder Maps [59] in PHENIX [55].

## Declaration of Competing Interest

The authors declare that they have no known competing financial interests or personal relationships that could have appeared to influence the work reported in this paper.

## Appendix Supplementary materials

Image, application 1
