# Supplementary material for: Fluorinated derivatives of pyridine-2,4-dicarboxylate are potent inhibitors of human 2-oxoglutarate dependent oxygenases
Source: J Fluor Chem. 2021 Jul;247:109804. doi: 10.1016/j.jfluchem.2021.109804 (PMC8223498; doi:10.1016/j.jfluchem.2021.109804)
Supplement: Supplementary file 1 [file mmc1.pdf]

Supplementary Information

Fluorinated derivatives of pyridine-2,4-dicarboxylate are potent inhibitors of human 2-oxoglutarate dependent oxygenases

Lennart Brewitz[1], Yu Nakashima[1,2], Anthony Tumber[1], Eidarus Salah[1], and Christopher J. Schofield[1,*]

[1]*Chemistry Research Laboratory, University of Oxford, 12 Mansfield Road, OX1 3TA, Oxford, United Kingdom.*

[2]*Present address: Institute of Natural Medicine, University of Toyama, 2630-Sugitani, 930-0194, Toyama, Japan.*

*christopher.schofield@chem.ox.ac.uk

Table of contents

1.	Supporting figures and tables	S2-S11
2.	References	S12
3.	^{1}H, ^{13}C, and ^{19}F NMR spectra of all novel compounds prepared for this study	S13-S27

1. Supporting figures and tables

Supporting Figure S1. Reactions catalyzed by the human 2OG oxygenases used in this work. (**a**) Aspartate/asparagine-β-hydroxylase (AspH) catalyzes the stereoselective C3 hydroxylation of Asp/Asn-residues which are part of specific disulfide isomers of epidermal growth factor-like domains (EGFDs) [1, 2]; (**b**) factor inhibiting the hypoxia inducible transcription factor-1α (FIH) catalyzes the stereoselective C3 hydroxylation of Asn/Asp/His/Ser/Leu-residues [3], such as, for example, the hydroxylation of Asn803 of the hypoxia inducible transcription factor-1α (HIF-1α) [4]; (**c**) JmjC lysine-specific N^{ε}-demethylase 4E (KDM4E, JMJD2E) catalyzes the N^{ε}-demethylation of histone 3 (H3) N^{ε}-di- and trimethylated Lys9 (H3K9me3/me2) via methyl-group oxidation with the formation of formaldehyde as a coproduct [5]; (**d**) ribosomal oxygenase 2 (RIOX2; MYC-induced nuclear antigen 53 or MINA53) catalyzes the stereoselective C3 hydroxylation of His39 of the 60S ribosomal protein L27a (RPL27A) [6].

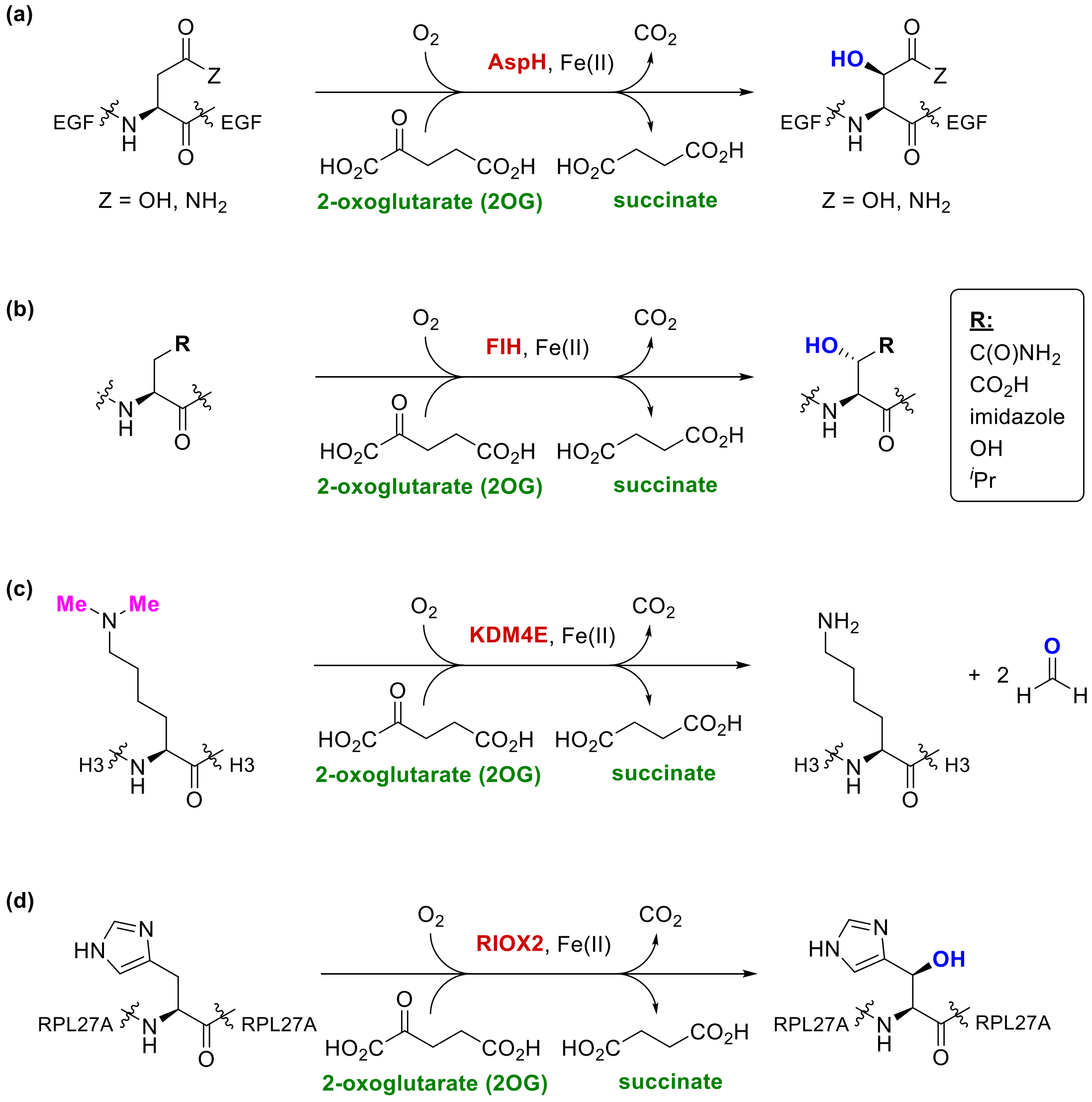

Supporting Figure S2. Robustness of the 2OG oxygenase SPE-MS inhibition assays. (**a**) Z'-factors [7] and (**b**) signal-to-noise ratios (S/N) for the 2OG oxygenase inhibition assay plates analyzed to determine IC_{50}-values. The Z'-factors >0.5 (grey line) indicate a stable and robust assay of high quality [7]. Z'-factors and S/N-ratios were determined according to the literature using Microsoft Excel [7]. Color code: AspH: orange circles; FIH: green boxes; KDM4E: blue triangles; RIOX2: pink diamonds.

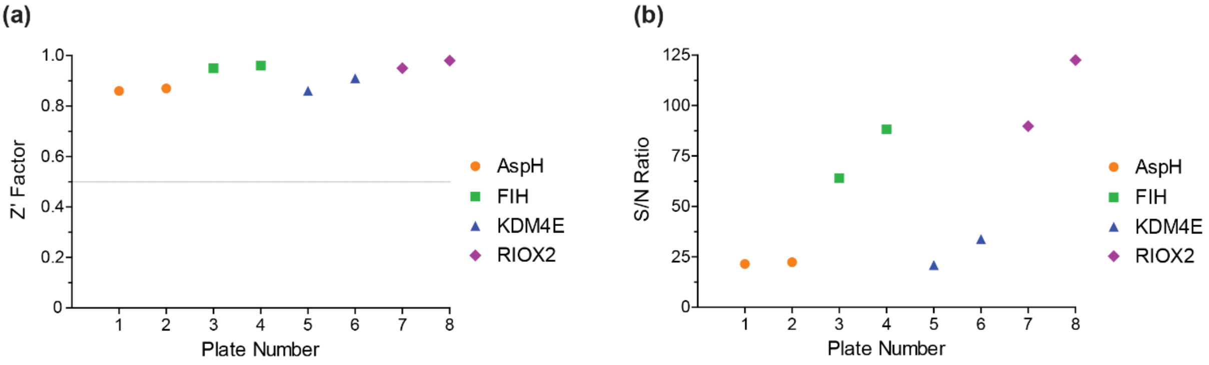

Supporting Figure S3. Representative dose-response curves used to determine IC_{50} values for 2,4-PDCA and its fluorinated/trifluoromethylated derivatives. SPE-MS 2OG oxygenase inhibition assays were performed as specified in the Experimental Section (4.4.). Inhibition curves are shown as a mean of two technical duplicates (n = 2; mean ± SD). The mean of two independent duplicates each composed of technical duplicates was used to determine IC_{50} values. Color code: pyridine-2,4-dicarboxylic acid (2,4-PDCA; **2**): blue inverse triangles; 3-fluoropyridine-2,4-dicarboxylic acid (**7**): green triangles; 5-fluoropyridine-2,4-dicarboxylic acid (**8**): red boxes; 3-trifluoromethylpyridine-2,4-dicarboxylic acid (**13**): orange circles; 5-trifluoromethylpyridine-2,4-dicarboxylic acid (**14**): black diamonds. (**a**) AspH; (**b**) FIH; (**c**) KDM4E; (**d**) RIOX2.

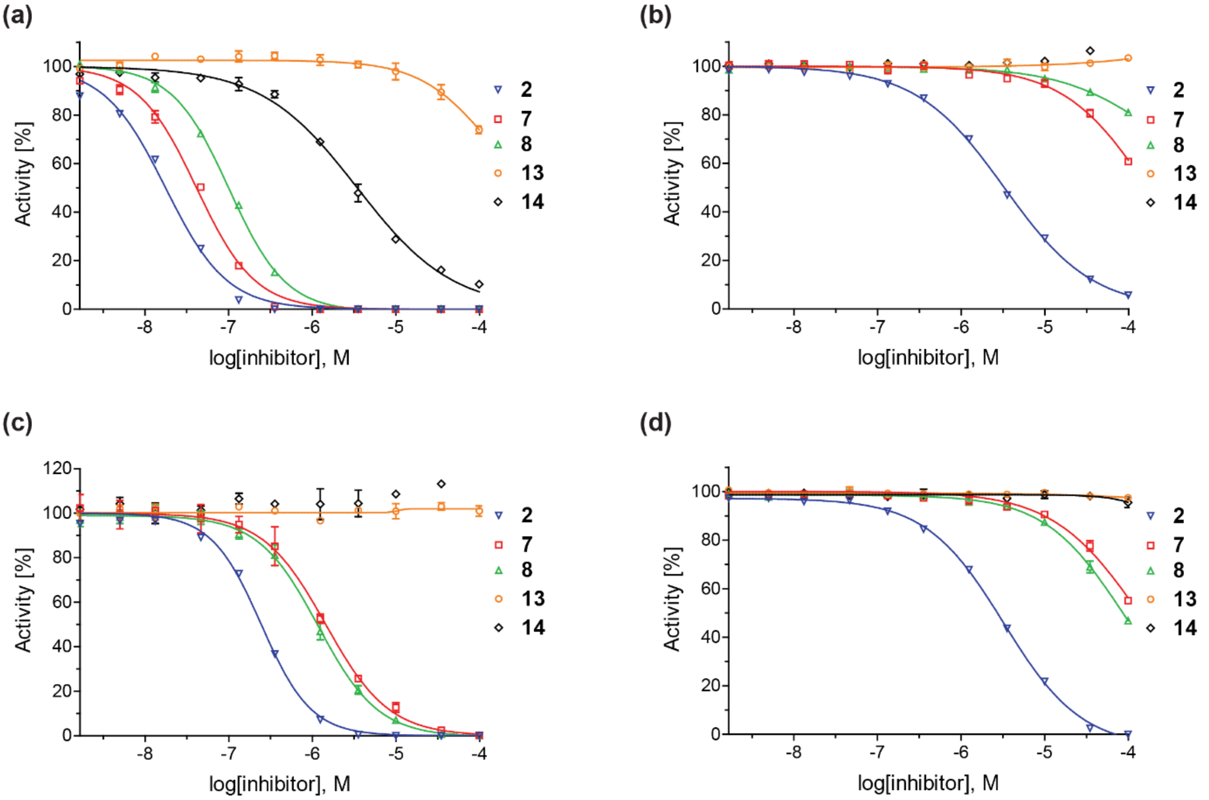

Supporting Figure S4. Sequence of the synthetic hFX-EGFD1$_{86\text{-}124}$-4Ser peptide used for AspH crystallization experiments. As reported, the sequence of the synthetic hFX-EGFD1$_{86\text{-}124}$-4Ser peptide [2] is based on the sequence of the EGFD1 of the reported AspH substrate human coagulation factor X (hFX) [8, 9]. Four hFX cysteine residues (Cys90$_{hFX}$, Cys95$_{hFX}$, Cys112$_{hFX}$, Cys121$_{hFX}$) have been substituted for serine residues (in blue) to avoid disulfide scrambling. The macrocycle formed by the Cys101$_{hFX}$–Cys110$_{hFX}$ disulfide bond (cystine sulfurs in green) is required for productive AspH substrate turnover [2]; The AspH hydroxylation site (Asp103$_{hFX}$) is in red.

hFX EGFD1$_{86\text{-}124}$-4Ser (hFX amino acids 86-124; Cys101$_{hFX}$- and Cys110$_{hFX}$-thiols form a disulfide bond):

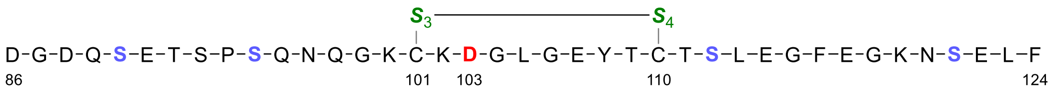

Supporting Figure S5. Views from a crystal structure of AspH complexed with 3-fluoropyridine-2,4-dicarboxylic acid (7), Mn, and the synthetic hFX-EGFD1$_{86\text{-}124}$-4Ser substrate peptide (AspH:7; PDB ID: 7MBI). Color code: grey: His$_6$-AspH$_{315\text{-}758}$; yellow: carbon-backbone of 3-fluoropyridine-2,4-dicarboxylic acid (**7**); lavender blue: Mn; green: carbon-backbone of the hFX-EGFD1$_{86\text{-}124}$-4Ser substrate peptide; red: oxygen; blue: nitrogen; pale green: fluorine.

(**a**) Overview of the AspH:**7** crystal structure (1.66 Å resolution); (**b**) representative OMIT electron density map (mF_o−DF_c) contoured to 2.5σ around the synthetic hFX-EGFD1$_{86\text{-}124}$-4Ser peptide of the AspH:**7** structure. The map reveals electron density for residues Gly99$_{hFX}$ to Thr111$_{hFX}$, including for the disulfide bridged (Cys101$_{hFX}$ and Cys110$_{hFX}$) ten-membered macrocycle. Note that the Asp103$_{hFX}$ side-chain carboxylate adopts two conformations, as observed in reported AspH:substrate complex structures [2, 10].

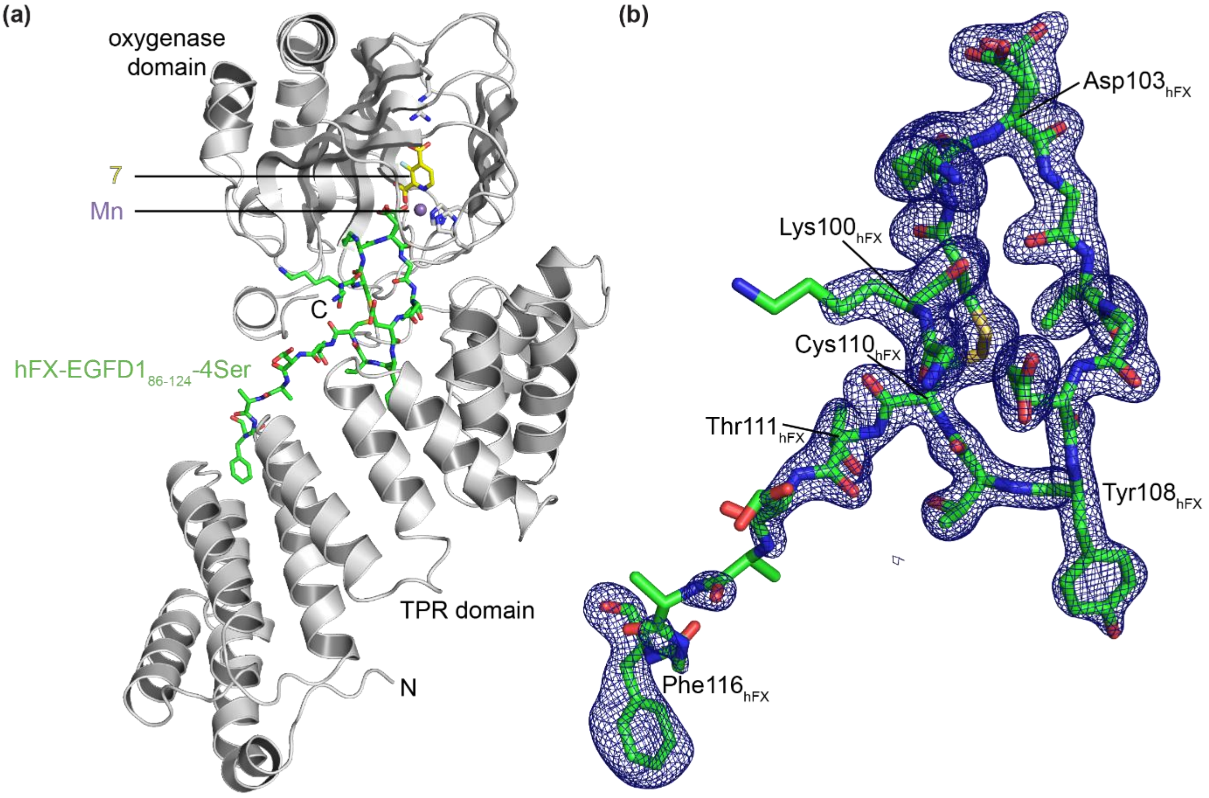

Supporting Figure S6. Views from a crystal structure of AspH complexed with 5-fluoropyridine-2,4-dicarboxylic acid (8), Mn, and the synthetic hFX-EGFD1$_{86-124}$-4Ser substrate peptide (AspH:8; PDB ID: 7MBJ). Color code: grey: His$_6$-AspH$_{315-758}$; salmon: carbon-backbone of 5-fluoropyridine-2,4-dicarboxylic acid (**8**); lavender blue: Mn; green: carbon-backbone of the hFX-EGFD1$_{86-124}$-4Ser substrate peptide; red: oxygen; blue: nitrogen; pale green: fluorine.

(**a**) Overview of a view of the AspH:**8** crystal structure (1.75 Å resolution). Note that two AspH molecules are present in the asymmetric unit. While both AspH molecules bind a Mn ion and **8**, only one of them binds to the synthetic hFX-EGFD1$_{86-124}$-4Ser peptide. The substrate-bound AspH molecule is shown; (**b**) representative OMIT electron density map (mF$_o$−DF$_c$) contoured to 2.5σ around the synthetic hFX-EGFD1$_{86-124}$-4Ser peptide of the AspH:**8** structure. The map reveals electron density for residues Gly99$_{hFX}$ to Phe116$_{hFX}$, including for the disulfide bridged (Cys101$_{hFX}$ and Cys110$_{hFX}$) ten-membered macrocycle. Note that the Asp103$_{hFX}$ side-chain carboxylate adopts two conformations, as observed in reported AspH:substrate complex structures [2, 10].

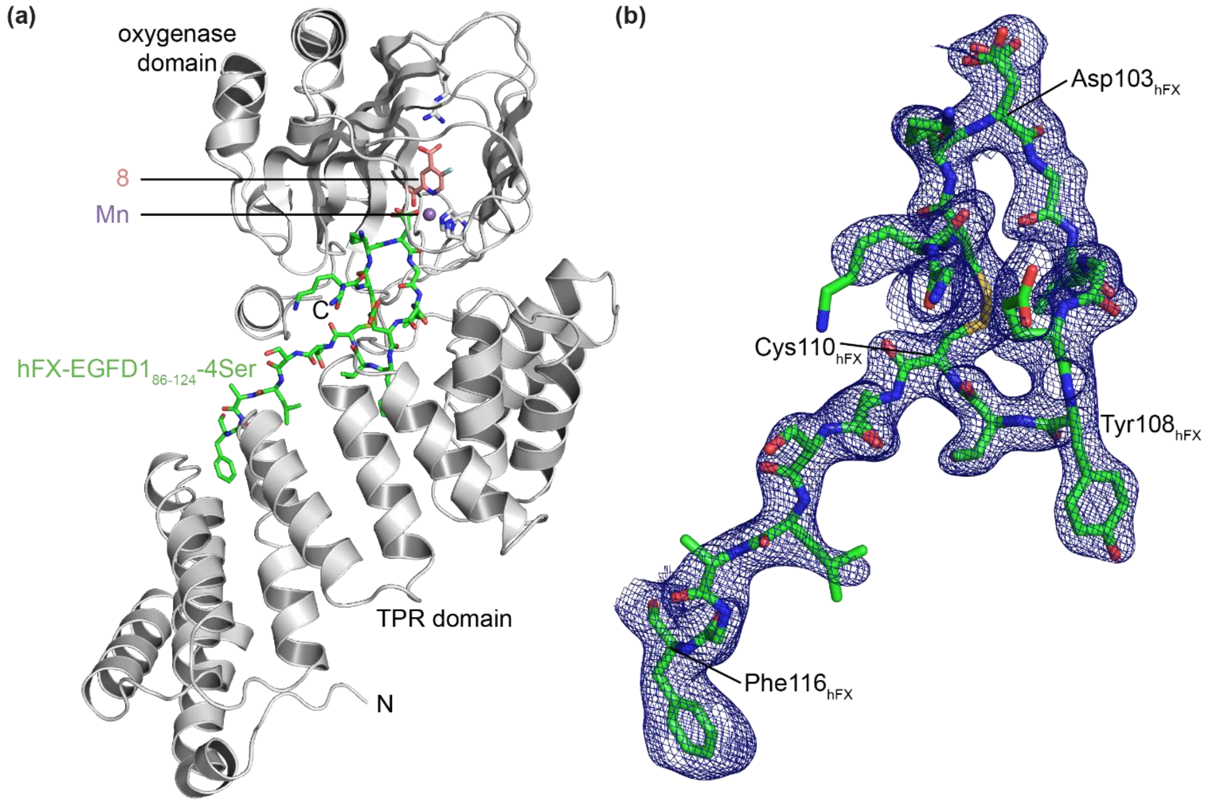

Supporting Figure S7. The substitution of pyridine-2,4-dicarboxylic acid by 3-fluoropyridine-2,4-dicarboxylic acid as an AspH ligand does not substantially change the conformations of AspH and of the synthetic hFX-EGFD1$_{86\text{-}124}$-4Ser substrate peptide. Color code: yellow: carbon-backbone of 3-fluoropyridine-2,4-dicarboxylic acid (**7**); cyan: carbon-backbone of pyridine-2,4-dicarboxylic acid (**2**); red: oxygen; blue: nitrogen; pale green: fluorine.

(**a**) Superimposition of a view from the AspH:**7** structure (grey: His$_6$-AspH$_{315\text{-}758}$; green: carbon-backbone of the hFX-EGFD1$_{86\text{-}124}$-4Ser substrate peptide; lavender blue: Mn; Supporting Figure S5) with one from the reported AspH:2,4-PDCA structure (bronze: His$_6$-AspH$_{315\text{-}758}$; brick red: carbon-backbone of the hFX-EGFD1$_{86\text{-}124}$-4Ser substrate peptide; violet: Mn; PDB ID: 5JTC) [11] reveals similar AspH conformations (Cα RMSD = 0.152 Å); (**b**) superimposition of the hFX-EGFD1$_{86\text{-}124}$-4Ser substrate peptide from the AspH:**7** structure (green: carbon-backbone of the hFX-EGFD1$_{86\text{-}124}$-4Ser substrate peptide) with the hFX-EGFD1$_{86\text{-}124}$-4Ser substrate peptide from the reported AspH:2,4-PDCA structure (brick red: carbon-backbone of the hFX-EGFD1$_{86\text{-}124}$-4Ser substrate peptide; PDB ID: 5JTC) [11] reveals similar peptide conformations (Cα RMSD = 0.266 Å).

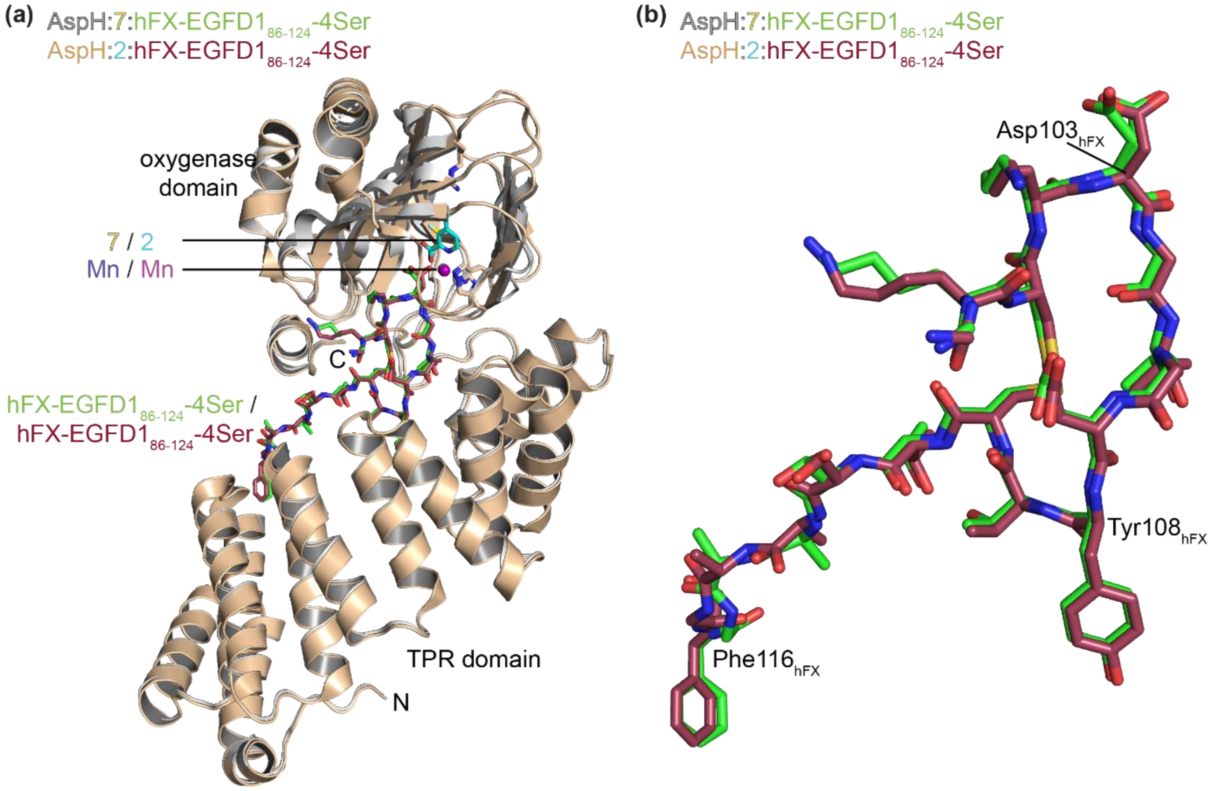

Supporting Figure S8. The substitution of pyridine-2,4-dicarboxylic acid by 5-fluoropyridine-2,4-dicarboxylic acid as an AspH ligand does not substantially change the conformations of AspH and of the synthetic hFX-EGFD1$_{86\text{-}124}$-4Ser substrate peptide. Color code: salmon: carbon-backbone of 5-fluoropyridine-2,4-dicarboxylic acid (**8**); cyan: carbon-backbone of pyridine-2,4-dicarboxylic acid (**2**); red: oxygen; blue: nitrogen; pale green: fluorine.

(**a**) Superimposition of a view from the AspH:**8** structure (grey: His$_6$-AspH$_{315\text{-}758}$; green: carbon-backbone of the hFX-EGFD1$_{86\text{-}124}$-4Ser substrate peptide; lavender blue: Mn; Supporting Figure S6) with one from the reported AspH:2,4-PDCA structure (bronze: His$_6$-AspH$_{315\text{-}758}$; brick red: carbon-backbone of the hFX-EGFD1$_{86\text{-}124}$-4Ser substrate peptide; violet: Mn; PDB ID: 5JTC) [11] reveals similar AspH conformations (Cα RMSD = 0.403 Å); (**b**) superimposition of the hFX-EGFD1$_{86\text{-}124}$-4Ser substrate peptide from the AspH:**8** structure (green: carbon-backbone of the hFX-EGFD1$_{86\text{-}124}$-4Ser substrate peptide) with the hFX-EGFD1$_{86\text{-}124}$-4Ser substrate peptide from the reported AspH:2,4-PDCA structure (brick red: carbon-backbone of the hFX-EGFD1$_{86\text{-}124}$-4Ser substrate peptide; PDB ID: 5JTC) [11] reveals similar peptide conformations (Cα RMSD = 0.188 Å).

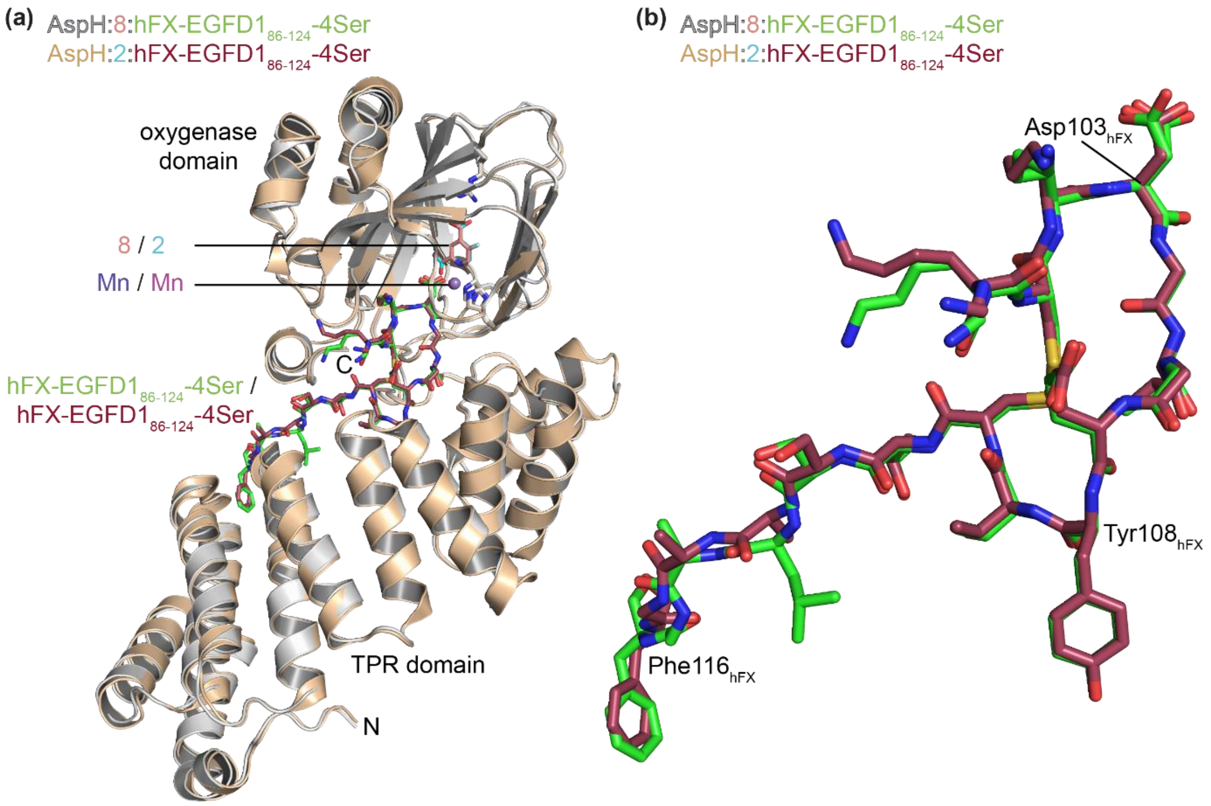

Supporting Figure S9. The substitution of 3-fluoropyridine-2,4-dicarboxylic acid by 5-fluoropyridine-2,4-dicarboxylic acid as an AspH ligand does not substantially change the conformations of AspH and of the synthetic hFX-EGFD1$_{86\text{-}124}$-4Ser substrate peptide. Color code: yellow: carbon-backbone of 3-fluoropyridine-2,4-dicarboxylic acid (**7**); salmon: carbon-backbone of 5-fluoropyridine-2,4-dicarboxylic acid (**8**); red: oxygen; blue: nitrogen; pale green: fluorine.

(**a**) Superimposition of a view from the AspH:**7** structure (grey: His$_6$-AspH$_{315\text{-}758}$; green: carbon-backbone of the hFX-EGFD1$_{86\text{-}124}$-4Ser substrate peptide; lavender blue: Mn; Supporting Figure S5) with one from the AspH:**8** structure (teal: His$_6$-AspH$_{315\text{-}758}$; deep blue: carbon-backbone of the hFX-EGFD1$_{86\text{-}124}$-4Ser substrate peptide; violet: Mn; Supporting Figure S6) reveals similar AspH conformations (Cα RMSD = 0.484 Å); (**d**) superimposition of the hFX-EGFD1$_{86\text{-}124}$-4Ser substrate peptide from the AspH:**7** structure (green: carbon-backbone of the hFX-EGFD1$_{86\text{-}124}$-4Ser substrate peptide) with the hFX-EGFD1$_{86\text{-}124}$-4Ser substrate peptide from the AspH:**8** structure (deep blue: carbon-backbone of the hFX-EGFD1$_{86\text{-}124}$-4Ser substrate peptide) reveals similar peptide conformations (Cα RMSD = 0.287 Å).

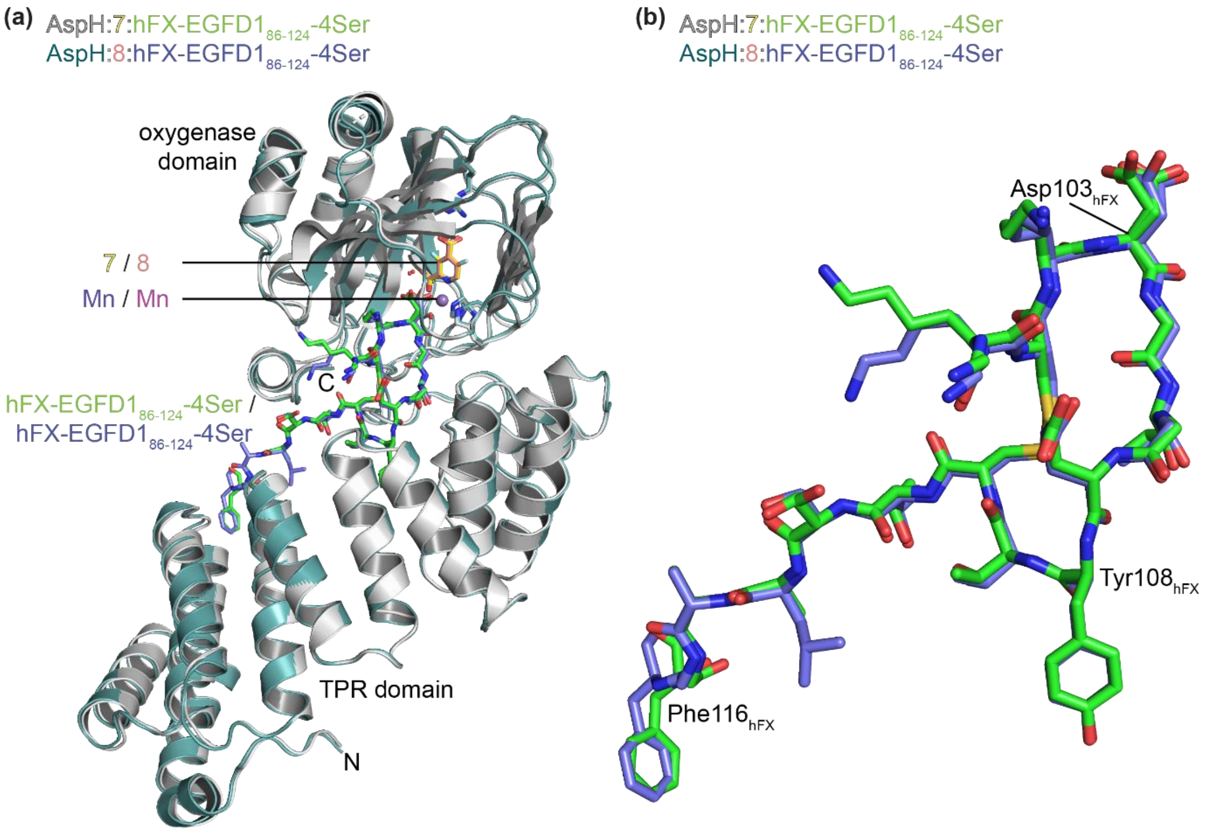

Supporting Table S1. Crystallization conditions, data collection, and refinement statistics for the AspH:2,4-PDCA derivative complexes.[a]

	$AspH_{315-758}$·Mn^{II}·**7**·hFX-EGFD1_{86-124}-4Ser (AspH:**7**)	$AspH_{315-758}$·Mn^{II}·**8**·hFX-EGFD1_{86-124}-4Ser (AspH:**8**)
**PDB ID**	7MBI	7MBJ
**Crystallization**		
Precipitation conditions	18 mg/mL His_6-$AspH_{315-758}$ (330 µM), 1 mM $MnCl_2$, 2 mM **7**, 100 mM HEPES, pH 7.0, 200 mM ammonium chloride, $20\%_{w/v}$ PEG6000, 726 µM hFX-EGFD1_{86-124}-4Ser	18 mg/mL His_6-$AspH_{315-758}$ (330 µM), 1 mM $MnCl_2$, 2 mM **8**, 200 mM sodium nitrate, $20\%_{w/v}$ PEG3350, 726 µM hFX-EGFD1_{86-124}-4Ser
**Data collection**		
Space group	$P2_12_12_1$	$P1$
Symmetry	orthorhombic	triclinic
Cell dimensions:		
a, *b*, *c* (Å)	49.70, 91.27, 122.63	49.35, 59.48, 95.54
α, β, γ (°)	90.00, 90.00, 90.00	104.26, 91.51, 92.76
X-Ray source[b]	Synchrotron (DLS I03)	Synchrotron (DLS I24)
Resolution (Å)[c]	46.06-1.66 (1.69-1.66)	55.42-1.75 (1.78-1.75)
R_{merge}	0.101 (4.209)	0.122 (2.408)
$I / \sigma I$	10.9 (0.3)	5.7 (0.5)
CC (1/2)	0.999 (0.312)	0.990 (0.290)
Total number of reflections	881881 (42262)	356642 (17963)
Total number unique reflections	66800 (3237)	102437 (5049)
Completeness (%)	100.0 (100.0)	96.6 (95.7)
Multiplicity	13.2 (13.1)	3.5 (3.6)
**Refinement**		
R_{work} / R_{free}	0.189 / 0.220	0.221 / 0.251
No. atoms:	7536	14556
B-factors (Å^2):	48.4	62.5
R.m.s. deviations:		
Bond lengths (Å)	0.015	0.005
Bond angles (°)	1.097	0.673

[a)]Experimental details are specified in the Supplementary Information (Section 6); [b)]DLS: Diamond Light Source; [c)]Values in parentheses are for highest-resolution shell.

2. References

[1] J. Stenflo, E. Holme, S. Lindstedt, N. Chandramouli, L.H. Tsai Huang, J.P. Tam, R.B. Merrifield, Hydroxylation of aspartic acid in domains homologous to the epidermal growth factor precursor is catalyzed by a 2-oxoglutarate-dependent dioxygenase, Proc. Natl. Acad. Sci. USA. 86(2) (1989) 444-447.

[2] I. Pfeffer, L. Brewitz, T. Krojer, S.A. Jensen, G.T. Kochan, N.J. Kershaw, K.S. Hewitson, L.A. McNeill, H. Kramer, M. Münzel, R.J. Hopkinson, U. Oppermann, P.A. Handford, M.A. McDonough, C.J. Schofield, Aspartate/asparagine-β-hydroxylase crystal structures reveal an unexpected epidermal growth factor-like domain substrate disulfide pattern, Nat. Commun. 10(1) (2019) 4910.

[3] H. Choi, A.P. Hardy, T.M. Leissing, R. Chowdhury, Y. Nakashima, W. Ge, M. Markoulides, J.S. Scotti, P.A. Gerken, H. Thorbjornsrud, D. Kang, S. Hong, J. Lee, M.A. McDonough, H. Park, C.J. Schofield, A human protein hydroxylase that accepts D-residues, Commun. Chem. 3(1) (2020) 52.

[4] K.S. Hewitson, L.A. McNeill, M.V. Riordan, Y.-M. Tian, A.N. Bullock, R.W. Welford, J.M. Elkins, N.J. Oldham, S. Bhattacharya, J.M. Gleadle, P.J. Ratcliffe, C.W. Pugh, C.J. Schofield, Hypoxia-inducible factor (HIF) asparagine hydroxylase is identical to factor inhibiting HIF (FIH) and is related to the cupin structural family, J. Biol. Chem. 277(29) (2002) 26351-26355.

[5] L. Hillringhaus, W.W. Yue, N.R. Rose, S.S. Ng, C. Gileadi, C. Loenarz, S.H. Bello, J.E. Bray, C.J. Schofield, U. Oppermann, Structural and evolutionary basis for the dual substrate selectivity of human KDM4 histone demethylase family, J. Biol. Chem. 286(48) (2011) 41616-41625.

[6] W. Ge, A. Wolf, T. Feng, C.-h. Ho, R. Sekirnik, A. Zayer, N. Granatino, M.E. Cockman, C. Loenarz, N.D. Loik, A.P. Hardy, T.D.W. Claridge, R.B. Hamed, R. Chowdhury, L. Gong, C.V. Robinson, D.C. Trudgian, M. Jiang, M.M. Mackeen, J.S. McCullagh, Y. Gordiyenko, A. Thalhammer, A. Yamamoto, M. Yang, P. Liu-Yi, Z. Zhang, M. Schmidt-Zachmann, B.M. Kessler, P.J. Ratcliffe, G.M. Preston, M.L. Coleman, C.J. Schofield, Oxygenase-catalyzed ribosome hydroxylation occurs in prokaryotes and humans, Nat. Chem. Biol. 8(12) (2012) 960-962.

[7] J.-H. Zhang, T.D.Y. Chung, K.R. Oldenburg, A simple statistical parameter for use in evaluation and validation of high throughput screening assays, J. Biomol. Screen. 4(2) (1999) 67-73.

[8] P. Fernlund, J. Stenflo, β-hydroxyaspartic acid in vitamin K-dependent proteins, J. Biol. Chem. 258(20) (1983) 12509-12512.

[9] B.A. McMullen, K. Fujikawa, W. Kisiel, T. Sasagawa, W.N. Howald, E.Y. Kwa, B. Weinstein, Complete amino acid sequence of the light chain of human blood coagulation factor X: evidence for identification of residue 63 as β-hydroxyaspartic acid, Biochemistry 22(12) (1983) 2875-2884.

[10] L. Brewitz, Y. Nakashima, C.J. Schofield, Synthesis of 2-oxoglutarate derivatives and their evaluation as cosubstrates and inhibitors of human aspartate/asparagine-β-hydroxylase, Chem. Sci. 12 (2021) 1327-1342.

[11] L. Brewitz, A. Tumber, I. Pfeffer, M.A. McDonough, C.J. Schofield, Aspartate/asparagine-β-hydroxylase: a high-throughput mass spectrometric assay for discovery of small molecule inhibitors, Sci. Rep. 10(1) (2020) 8650.

3. ^{1}H, ^{13}C, and ^{19}F NMR spectra of all novel compounds prepared for this study

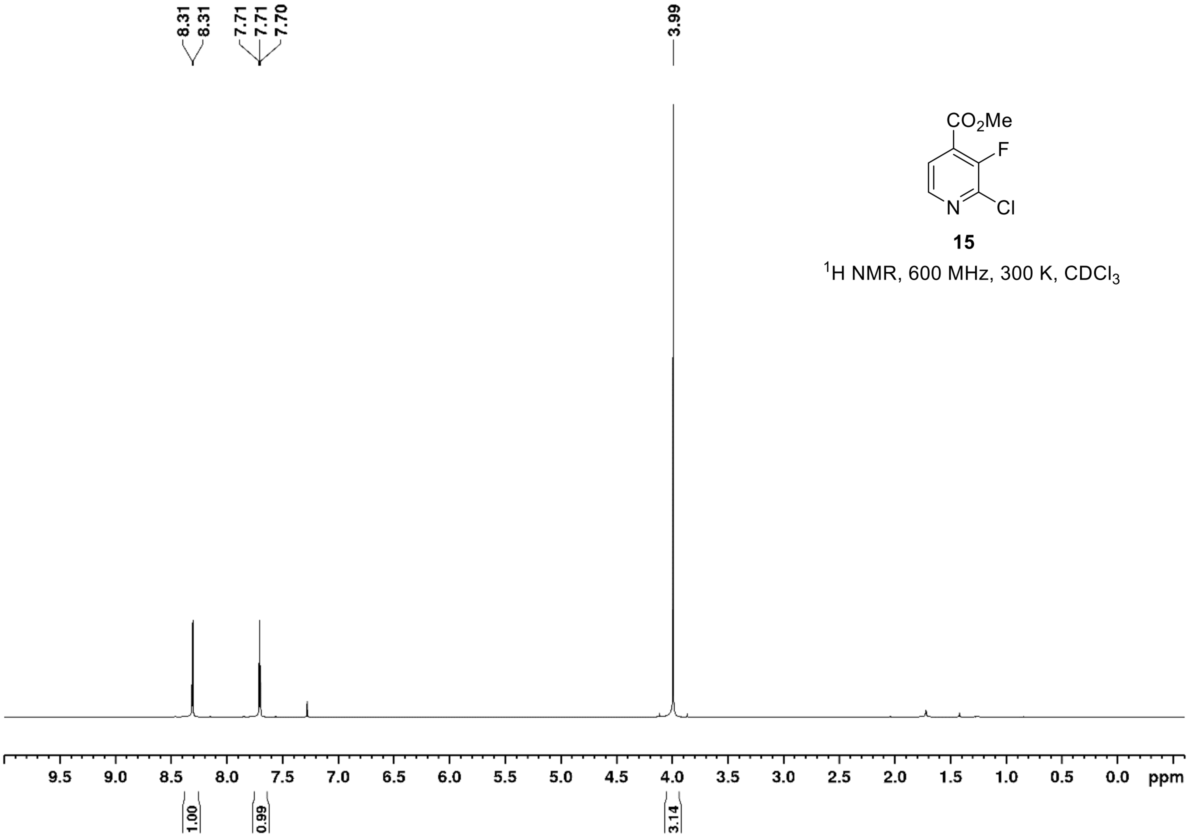

15

^{1}H NMR, 600 MHz, 300 K, $CDCl_3$

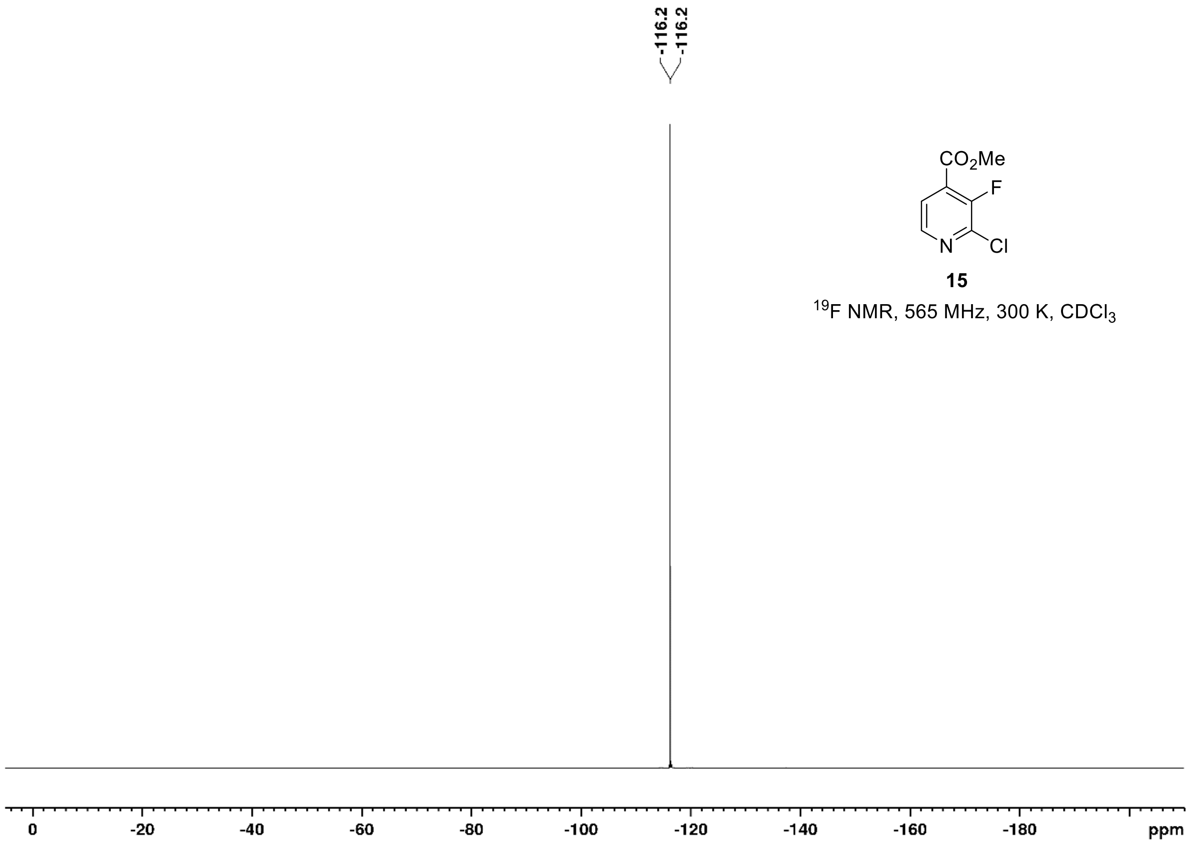

15

^{19}F NMR, 565 MHz, 300 K, $CDCl_3$

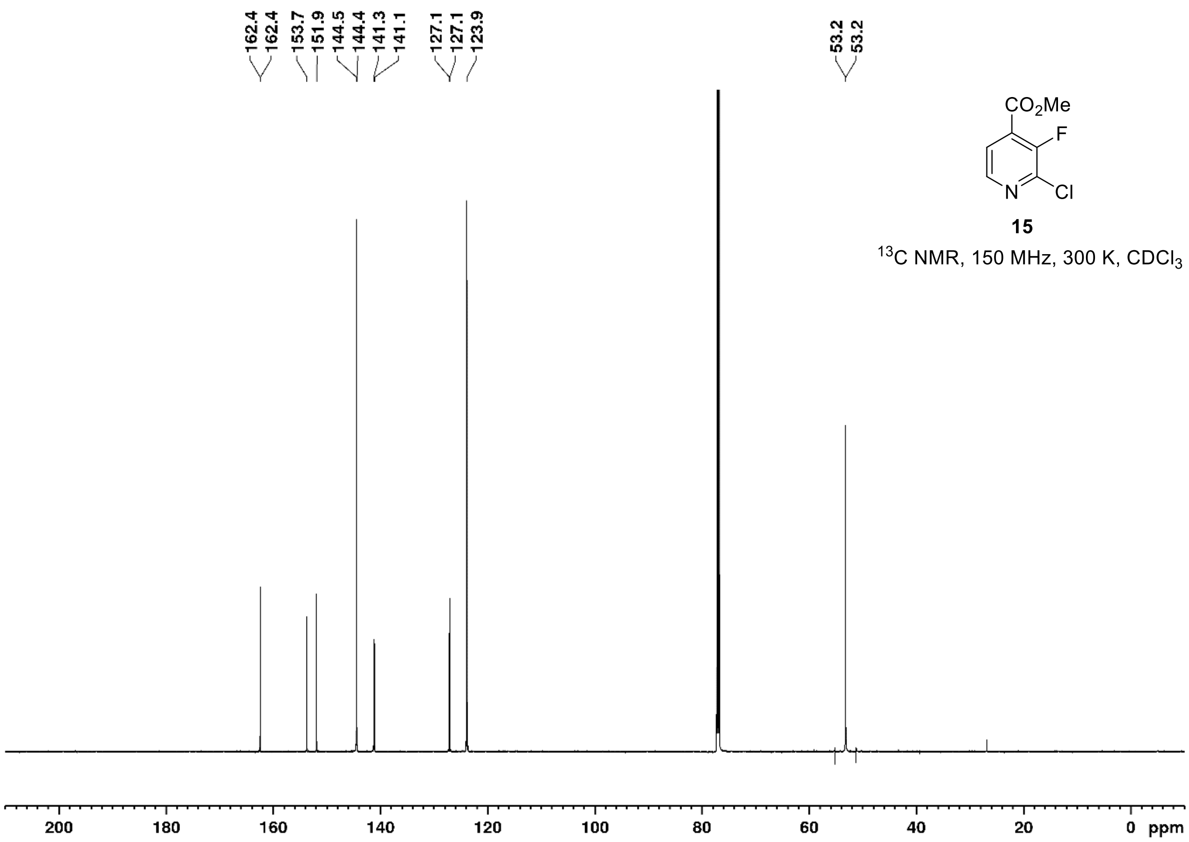

15

^{13}C NMR, 150 MHz, 300 K, $CDCl_3$

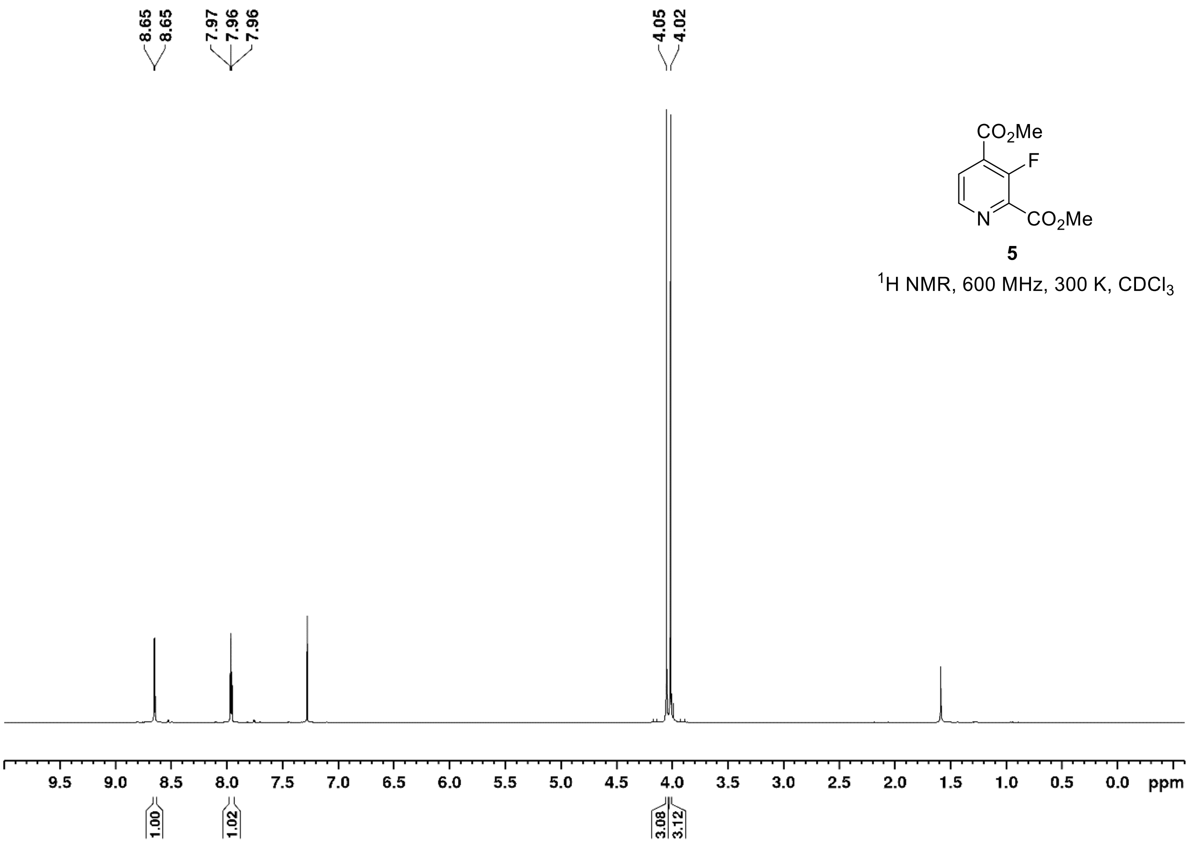

5

^{1}H NMR, 600 MHz, 300 K, $CDCl_3$

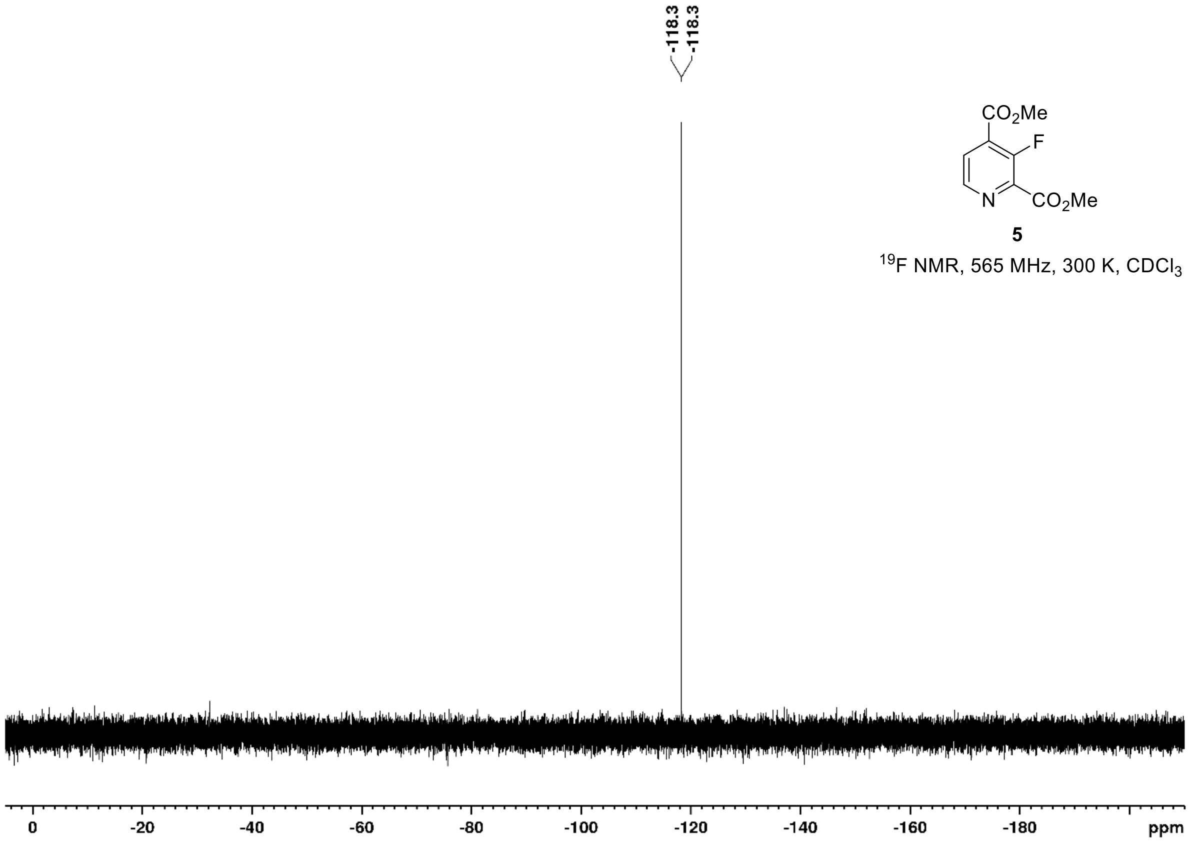

-118.3
-118.3
CO2Me
F
N
CO2Me
5
19F NMR, 565 MHz, 300 K, CDCl3
0
-20
-40
-60
-80
-100
-120
-140
-160
-180
ppm

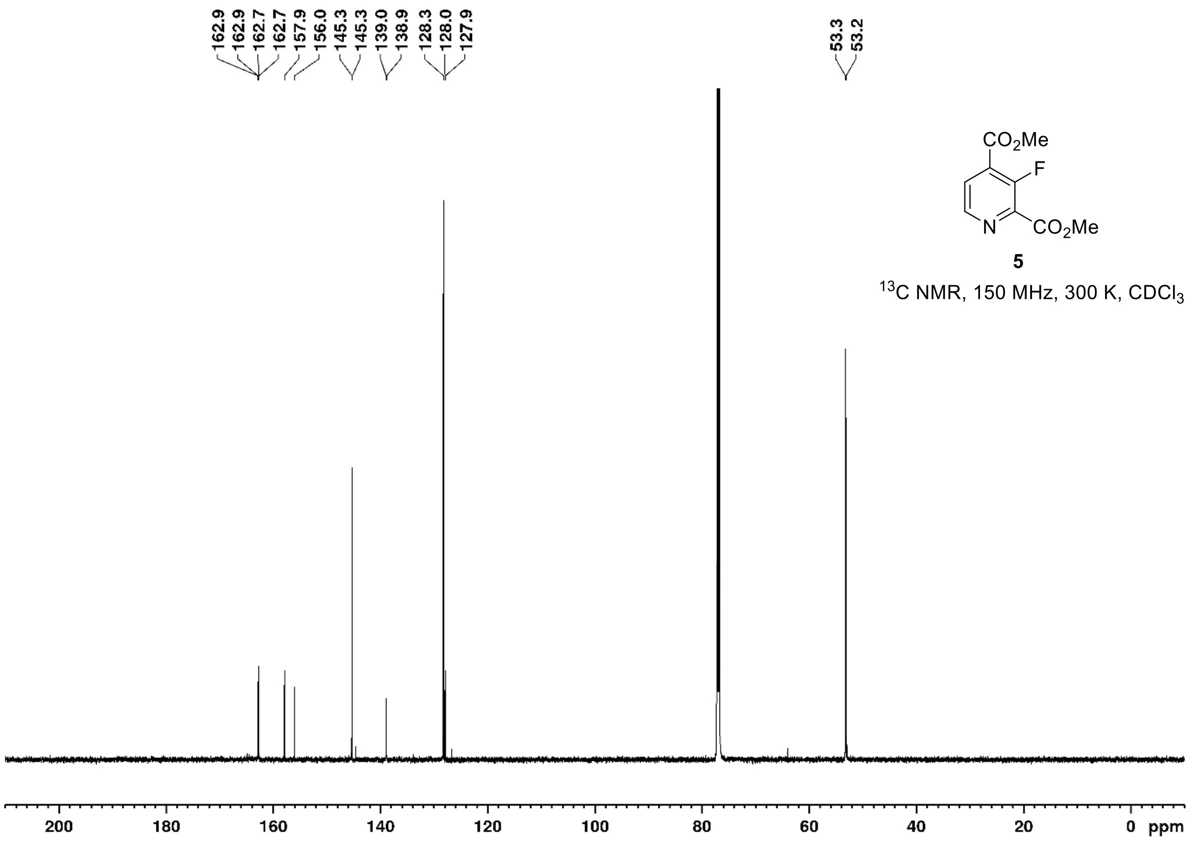

162.9
162.9
162.7
162.7
157.9
156.0
145.3
145.3
139.0
138.9
128.3
128.0
127.9
53.3
53.2
CO2Me
F
N
CO2Me
5
13C NMR, 150 MHz, 300 K, CDCl3
200
180
160
140
120
100
80
60
40
20
0 ppm

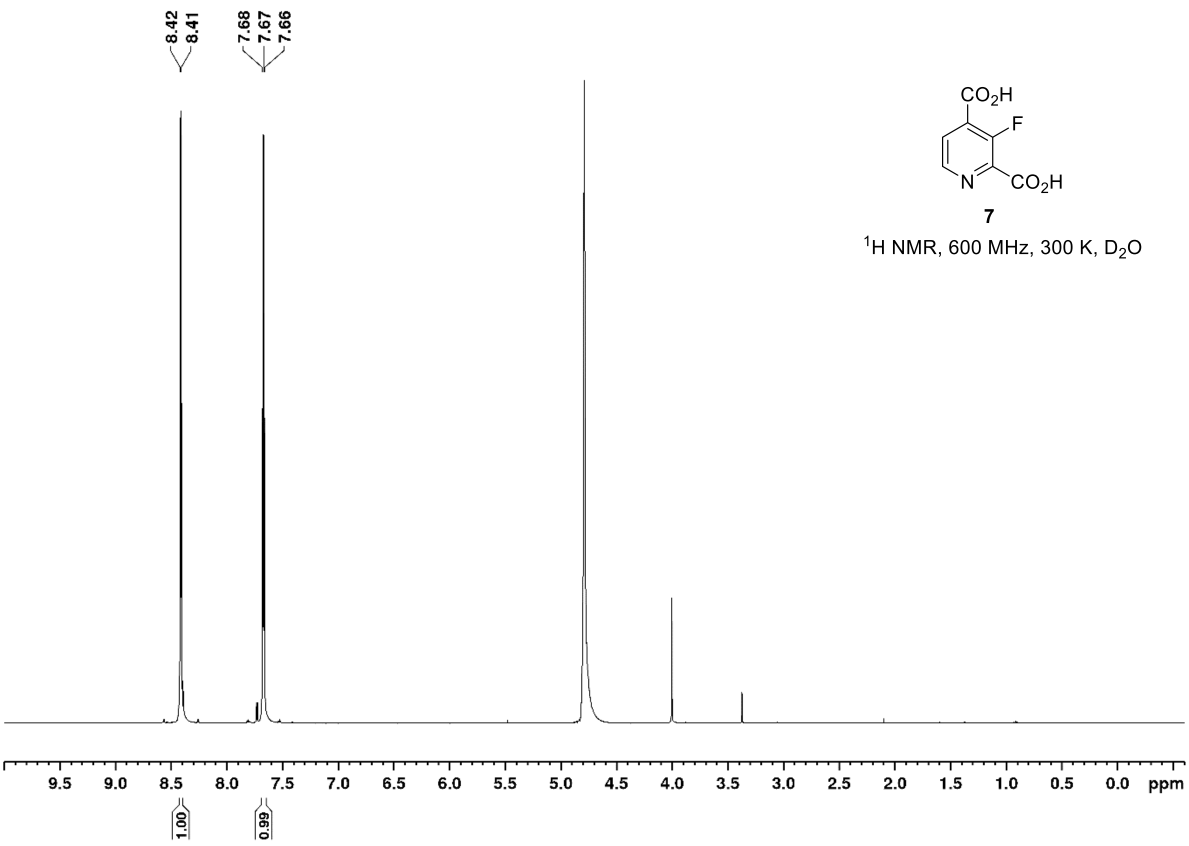
8.42
8.41
7.68
7.67
7.66
CO2H
F
N
CO2H
7
1H NMR, 600 MHz, 300 K, D2O
9.5 9.0 8.5 8.0 7.5 7.0 6.5 6.0 5.5 5.0 4.5 4.0 3.5 3.0 2.5 2.0 1.5 1.0 0.5 0.0 ppm
1.00
0.99

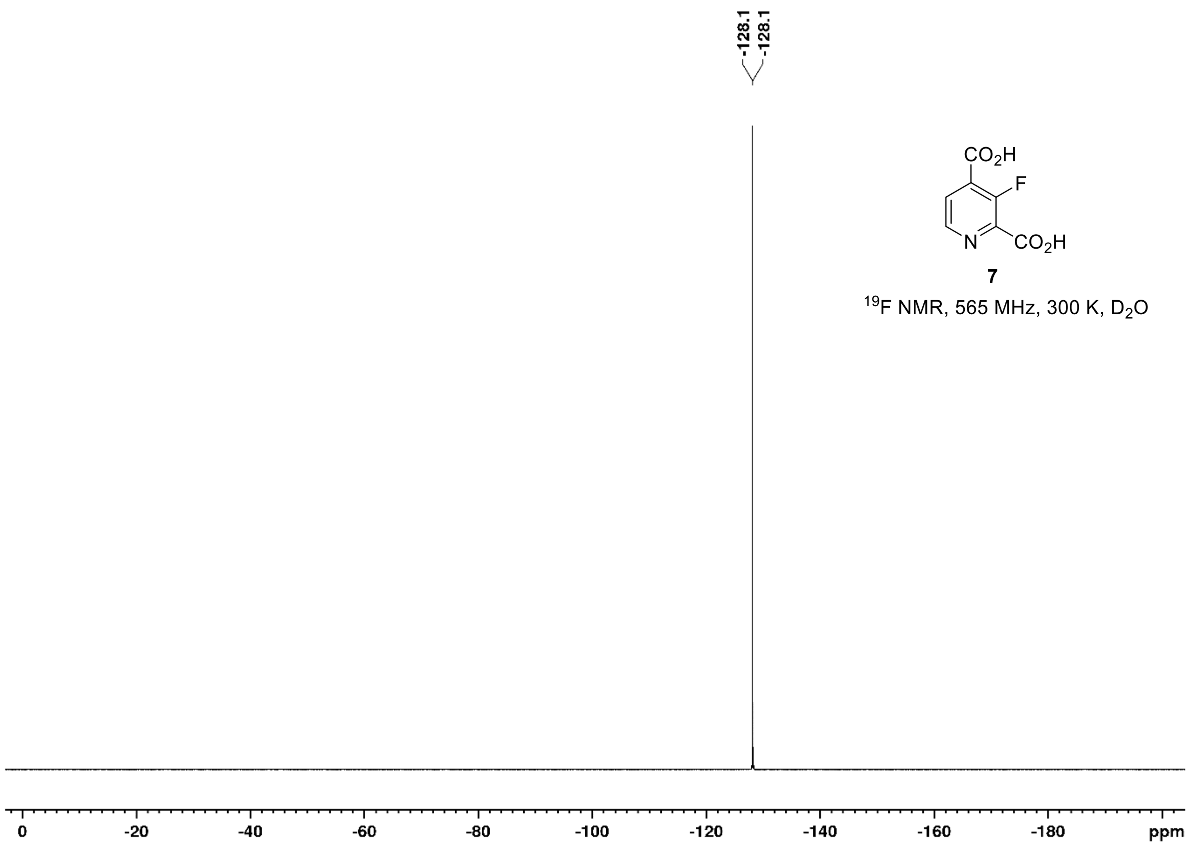
-128.1
-128.1
CO2H
F
N
CO2H
7
19F NMR, 565 MHz, 300 K, D2O
0 -20 -40 -60 -80 -100 -120 -140 -160 -180 ppm

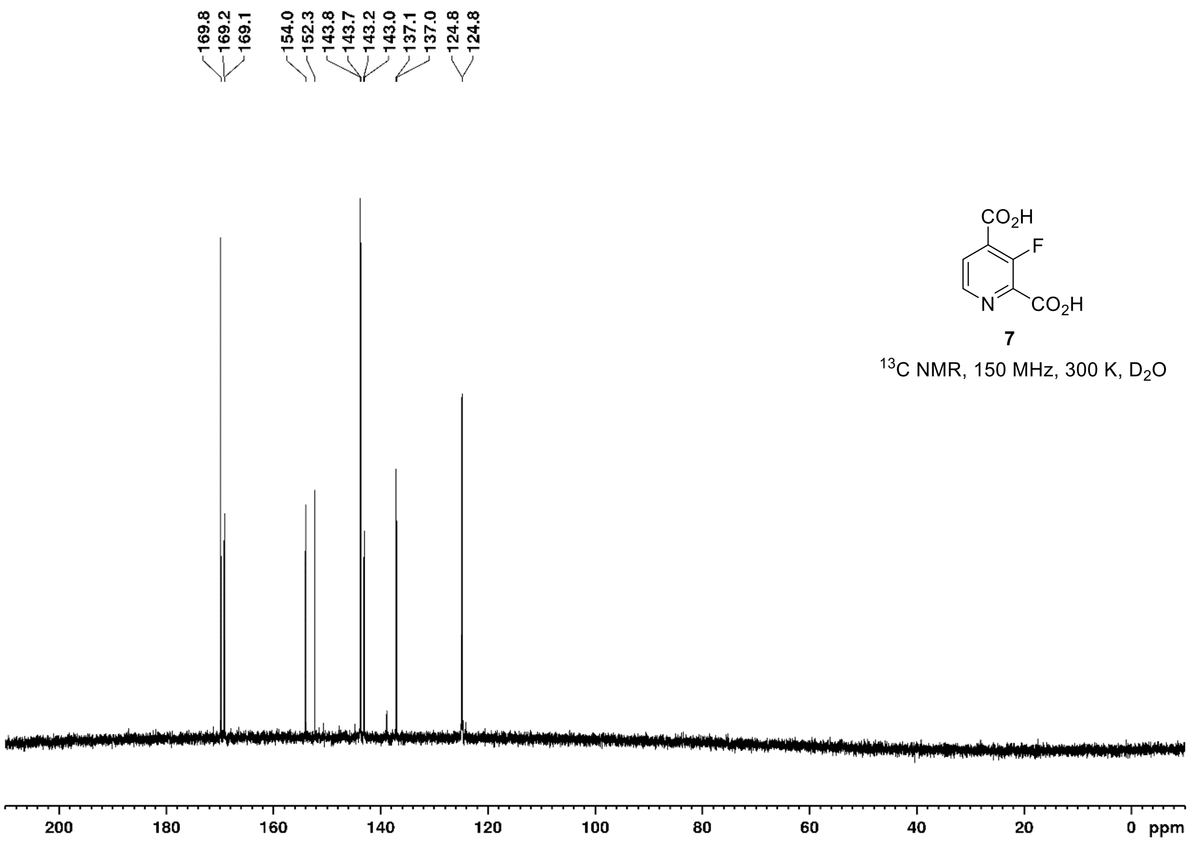

7

^{13}C NMR, 150 MHz, 300 K, D_2O

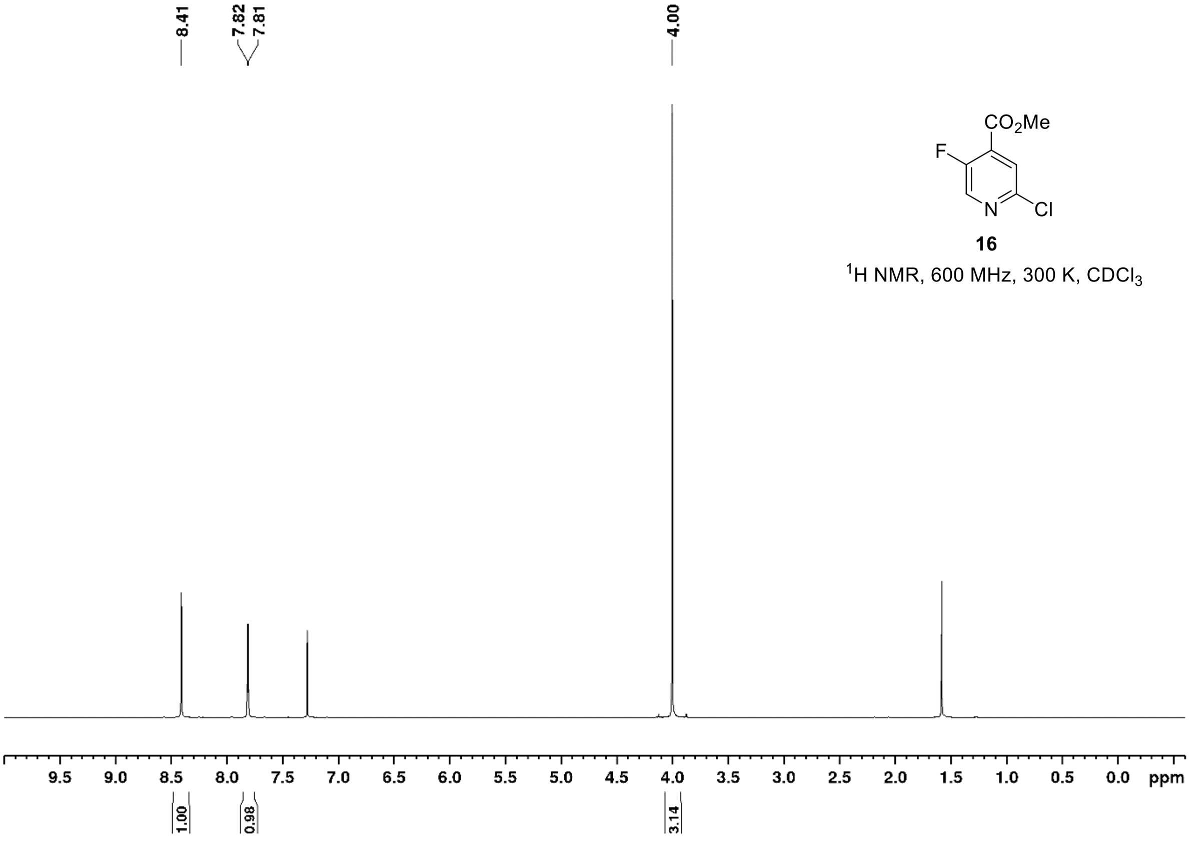

16

^{1}H NMR, 600 MHz, 300 K, $CDCl_3$

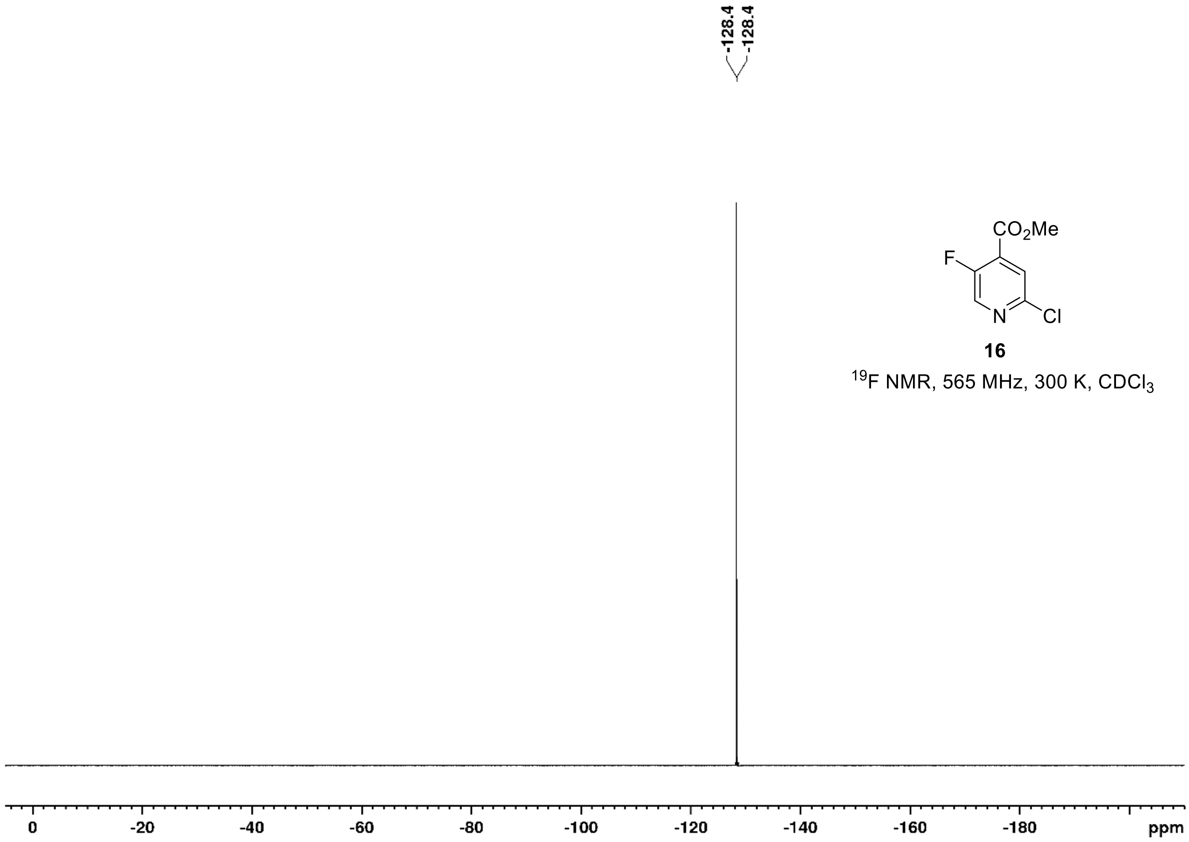
-128.4
-128.4
CO2Me
F
N
Cl
16
19F NMR, 565 MHz, 300 K, CDCl3
0
-20
-40
-60
-80
-100
-120
-140
-160
-180
ppm

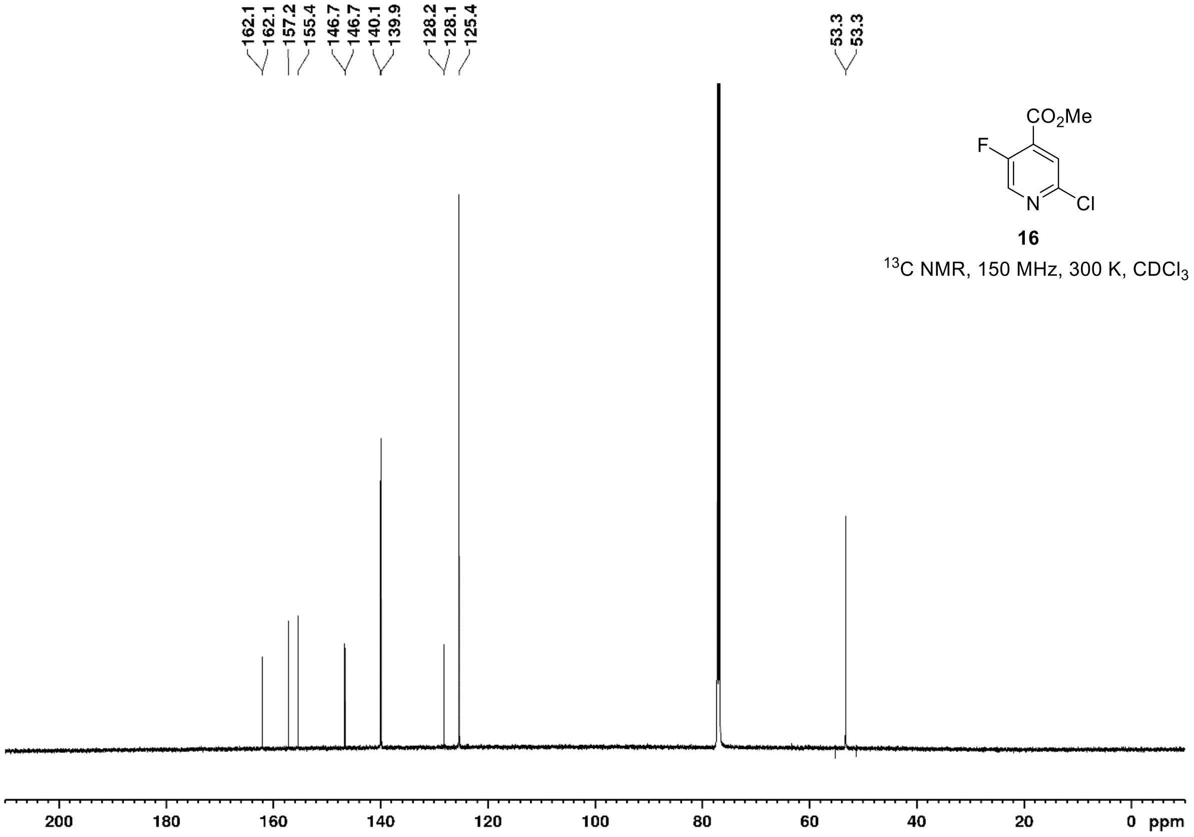
162.1
162.1
157.2
155.4
146.7
146.7
140.1
139.9
128.2
128.1
125.4
53.3
53.3
CO2Me
F
N
Cl
16
13C NMR, 150 MHz, 300 K, CDCl3
200
180
160
140
120
100
80
60
40
20
0 ppm

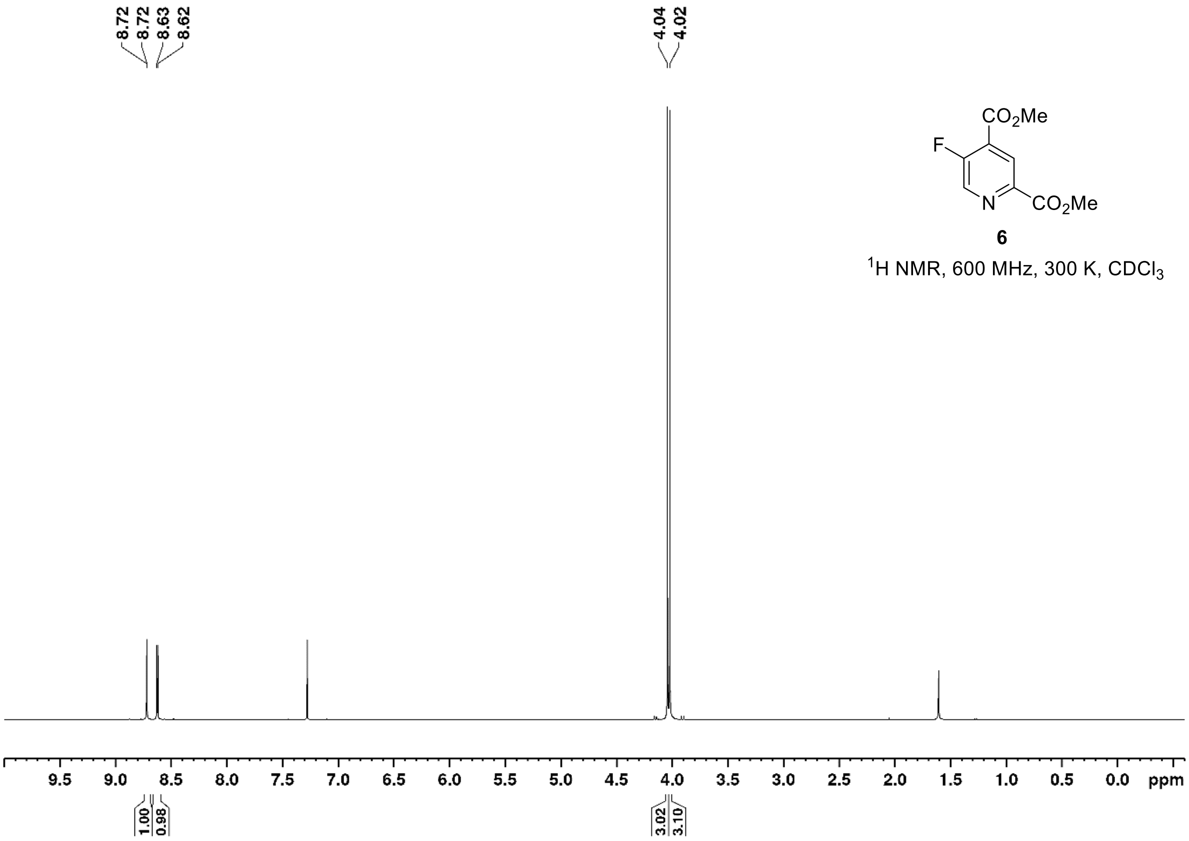

6

^{1}H NMR, 600 MHz, 300 K, $CDCl_3$

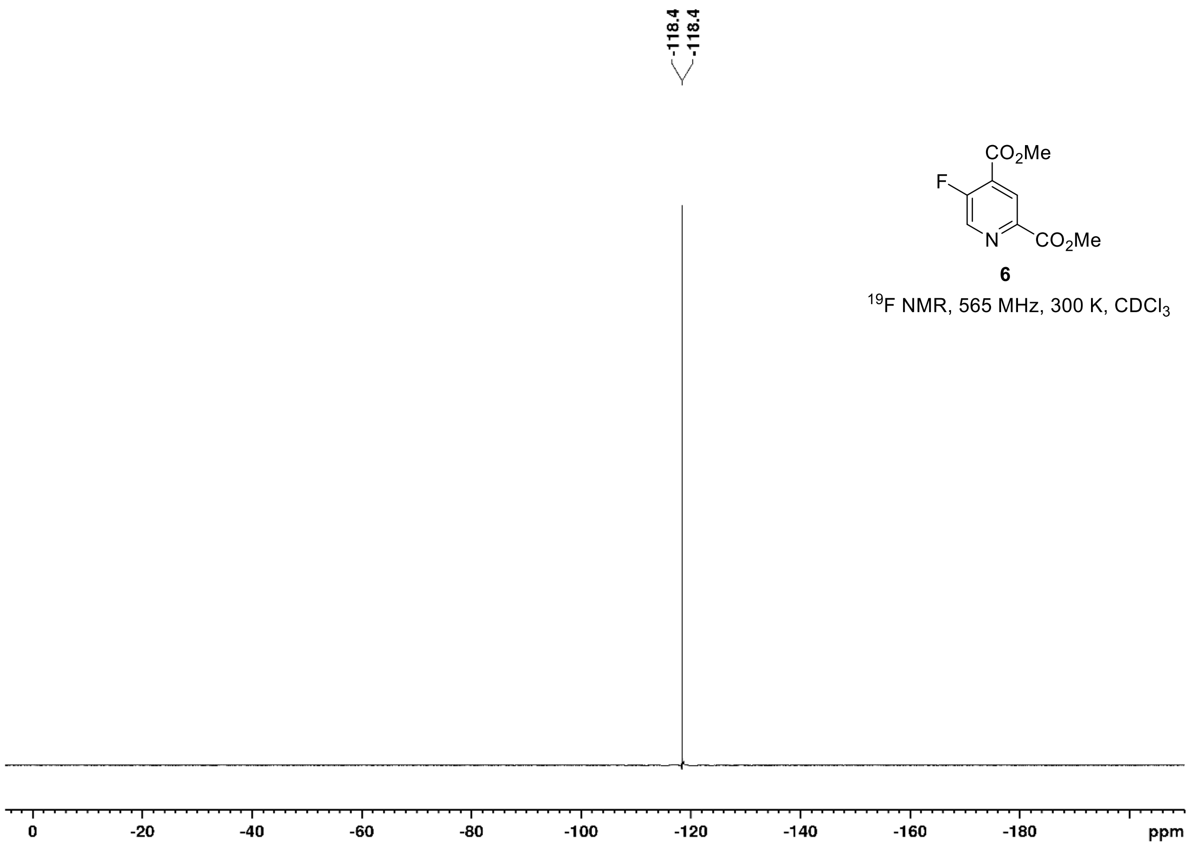

6

^{19}F NMR, 565 MHz, 300 K, $CDCl_3$

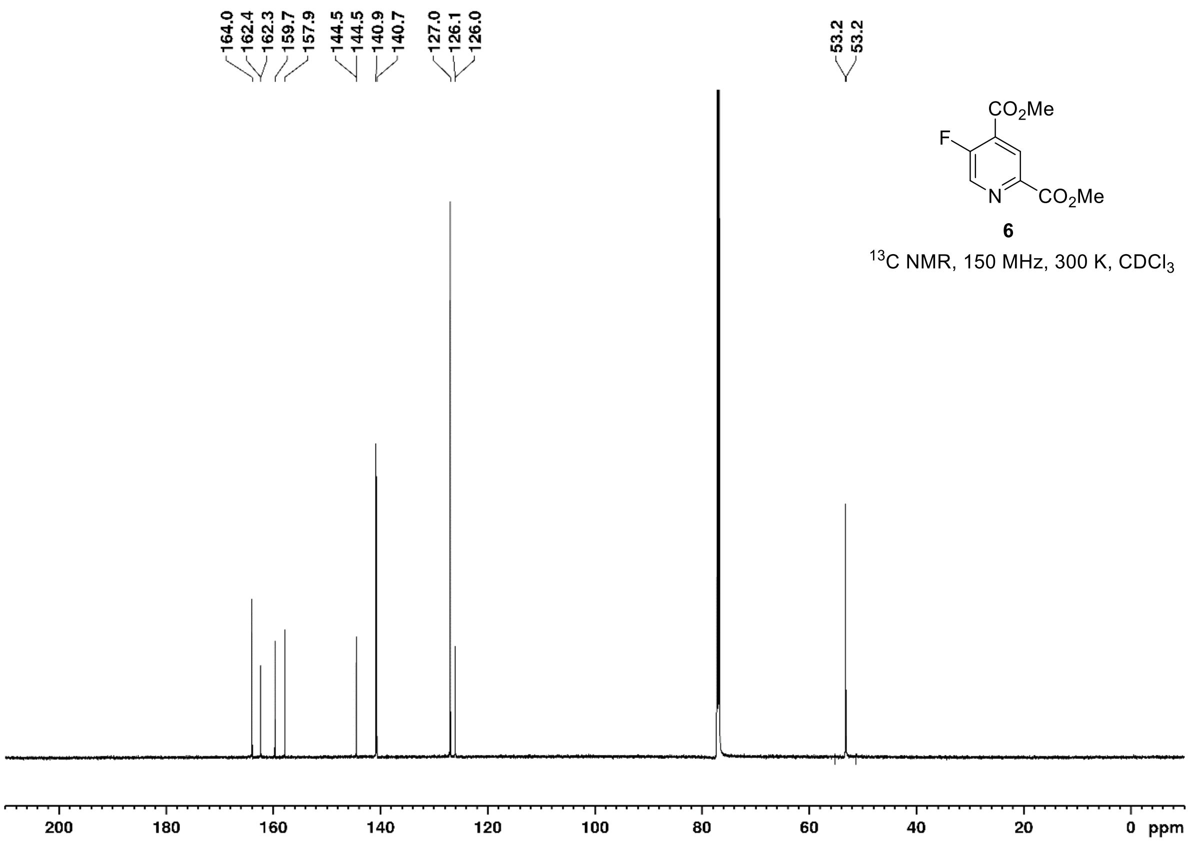
164.0
162.4
162.3
159.7
157.9
144.5
144.5
140.9
140.7
127.0
126.1
126.0
53.2
53.2
CO2Me
F
N
CO2Me
6
13C NMR, 150 MHz, 300 K, CDCl3
200
180
160
140
120
100
80
60
40
20
0 ppm

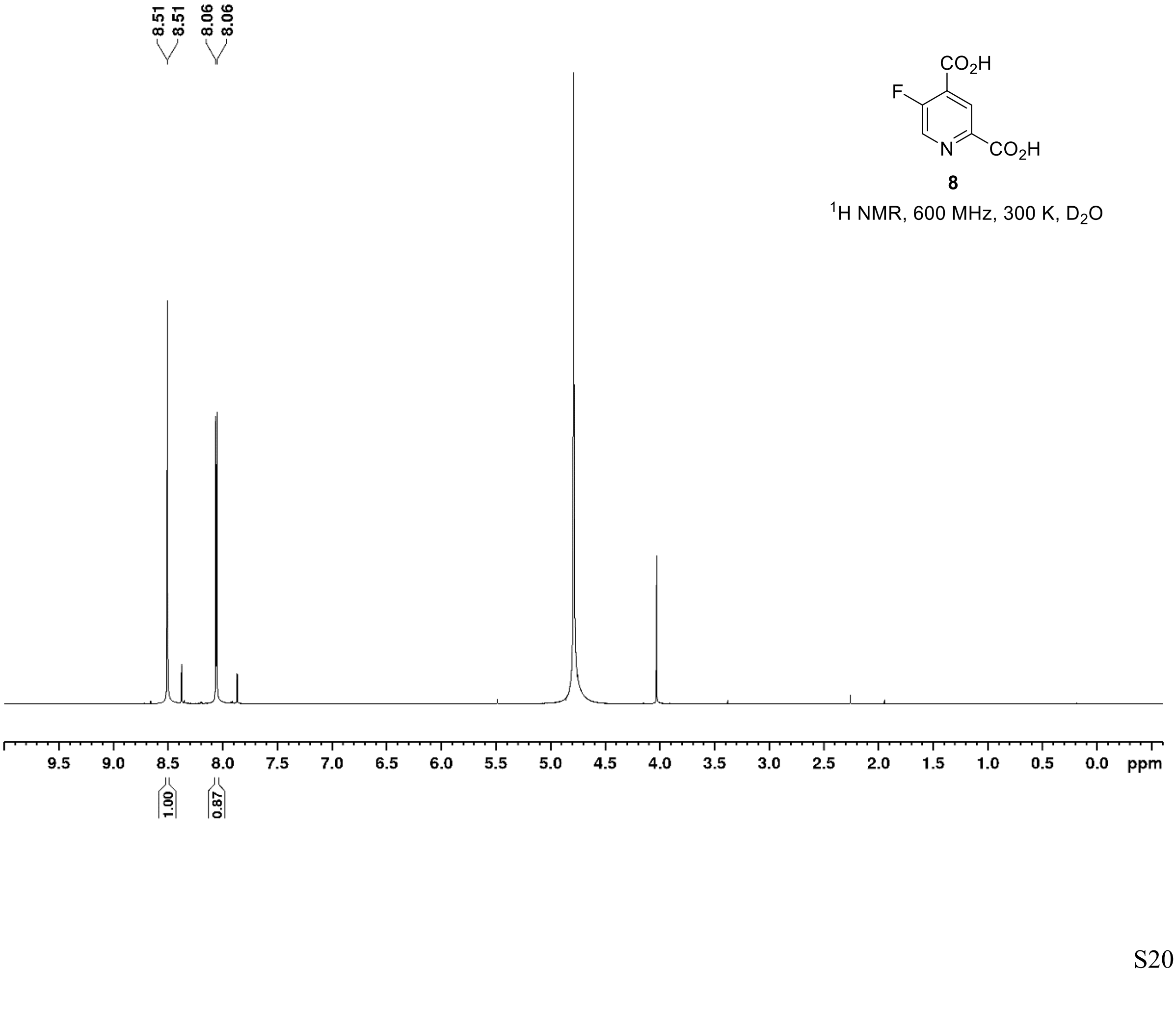
8.51
8.51
8.06
8.06
CO2H
F
N
CO2H
8
1H NMR, 600 MHz, 300 K, D2O
9.5
9.0
8.5
8.0
7.5
7.0
6.5
6.0
5.5
5.0
4.5
4.0
3.5
3.0
2.5
2.0
1.5
1.0
0.5
0.0
ppm
1.00
0.87

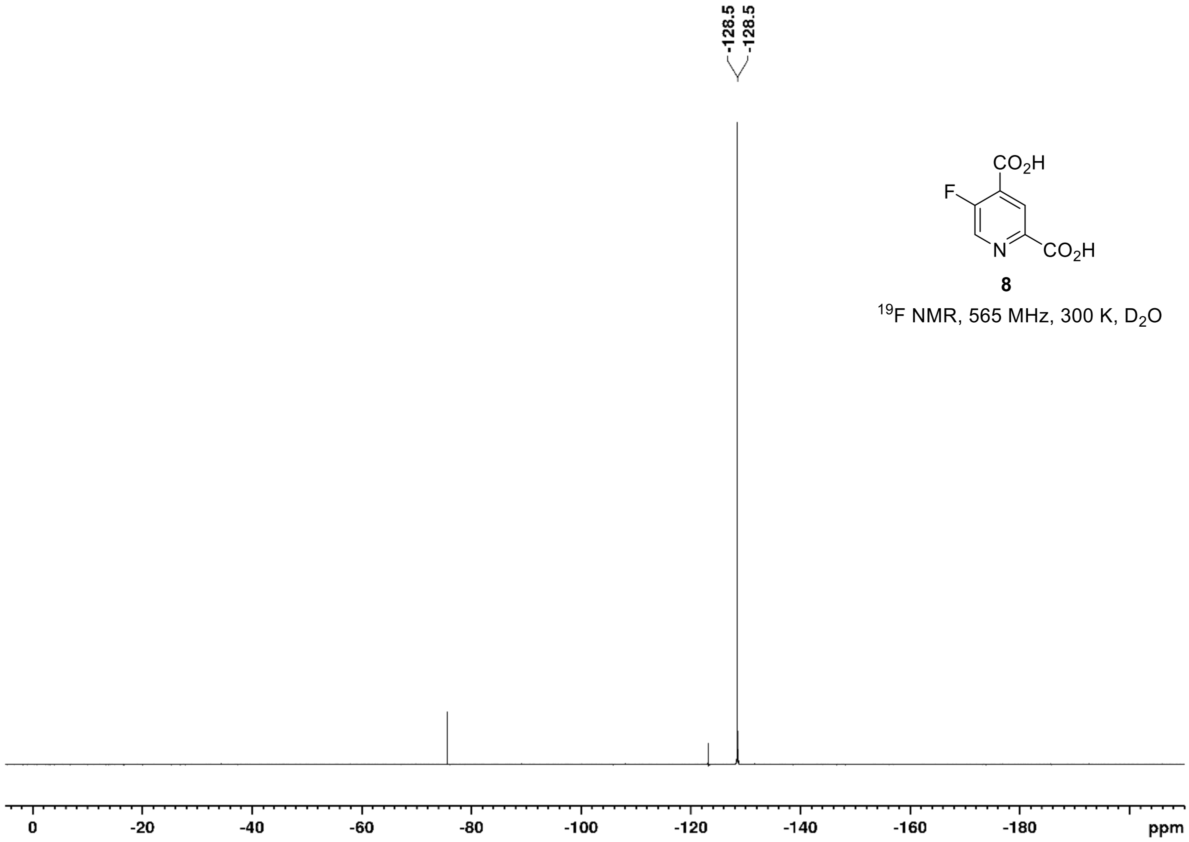
-128.5
-128.5
CO2H
F
N
CO2H
8
19F NMR, 565 MHz, 300 K, D2O
0 -20 -40 -60 -80 -100 -120 -140 -160 -180 ppm

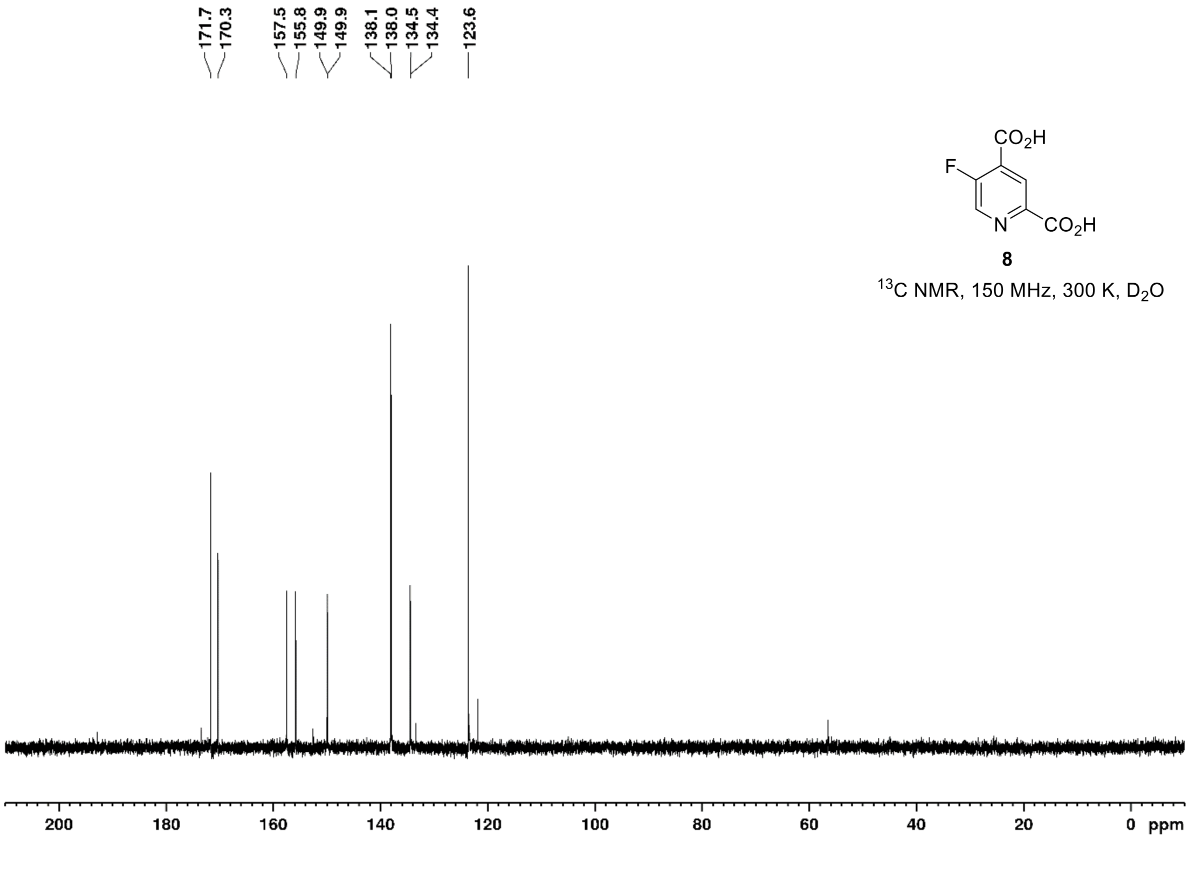
171.7
170.3
157.5
155.8
149.9
149.9
138.1
138.0
134.5
134.4
123.6
CO2H
F
N
CO2H
8
13C NMR, 150 MHz, 300 K, D2O
200 180 160 140 120 100 80 60 40 20 0 ppm

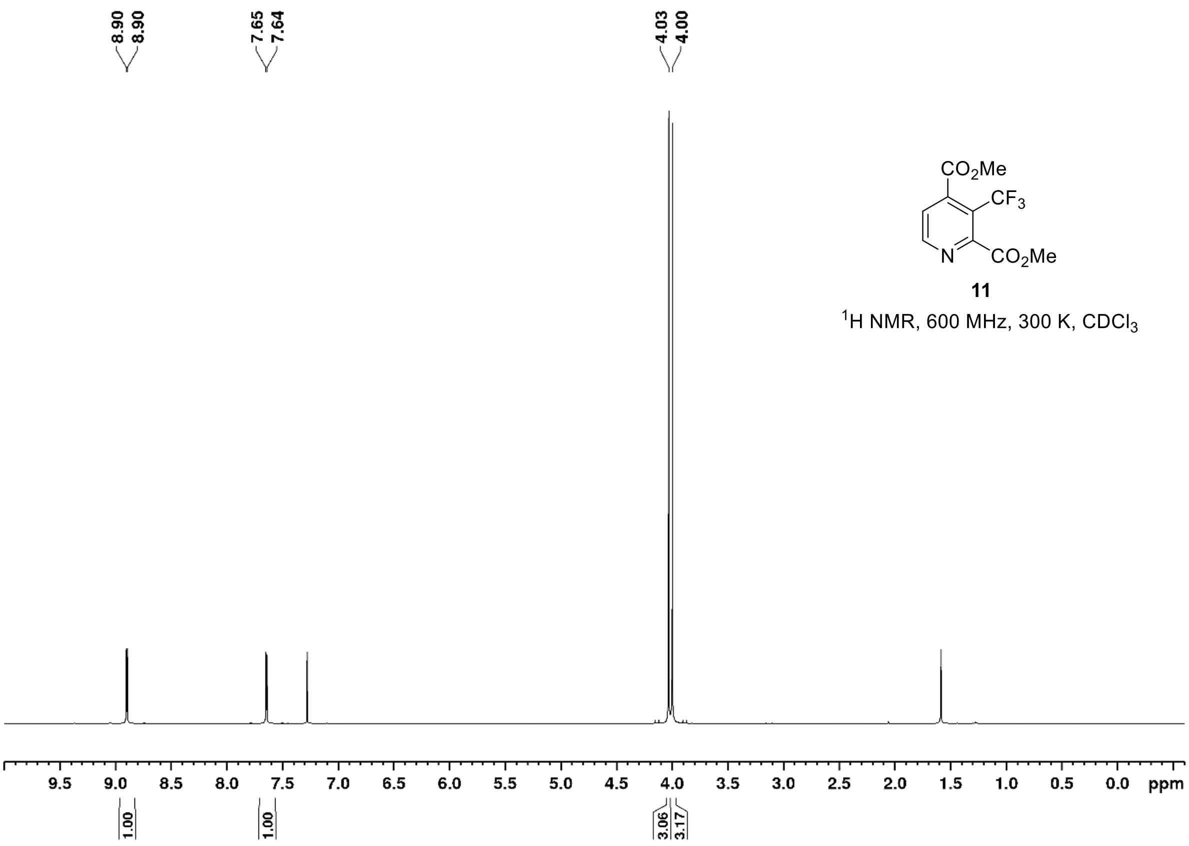

11

^{1}H NMR, 600 MHz, 300 K, $CDCl_3$

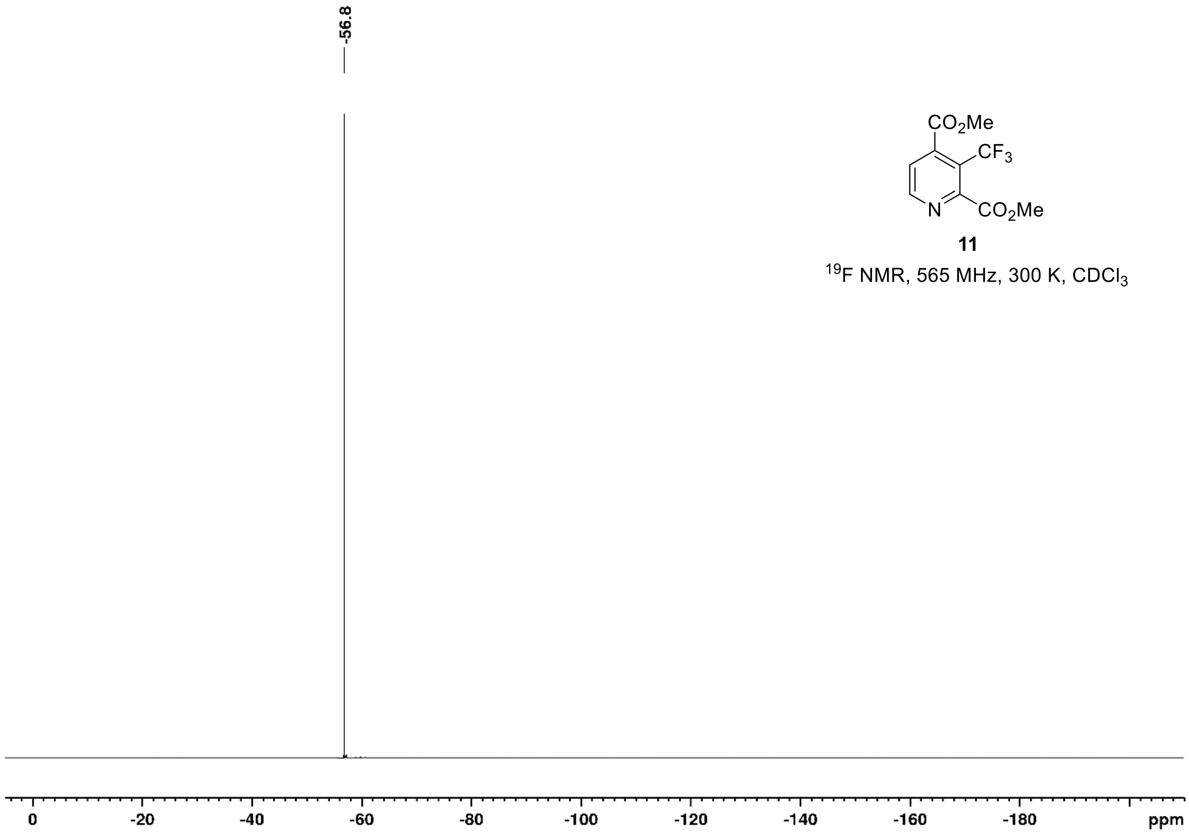

11

^{19}F NMR, 565 MHz, 300 K, $CDCl_3$

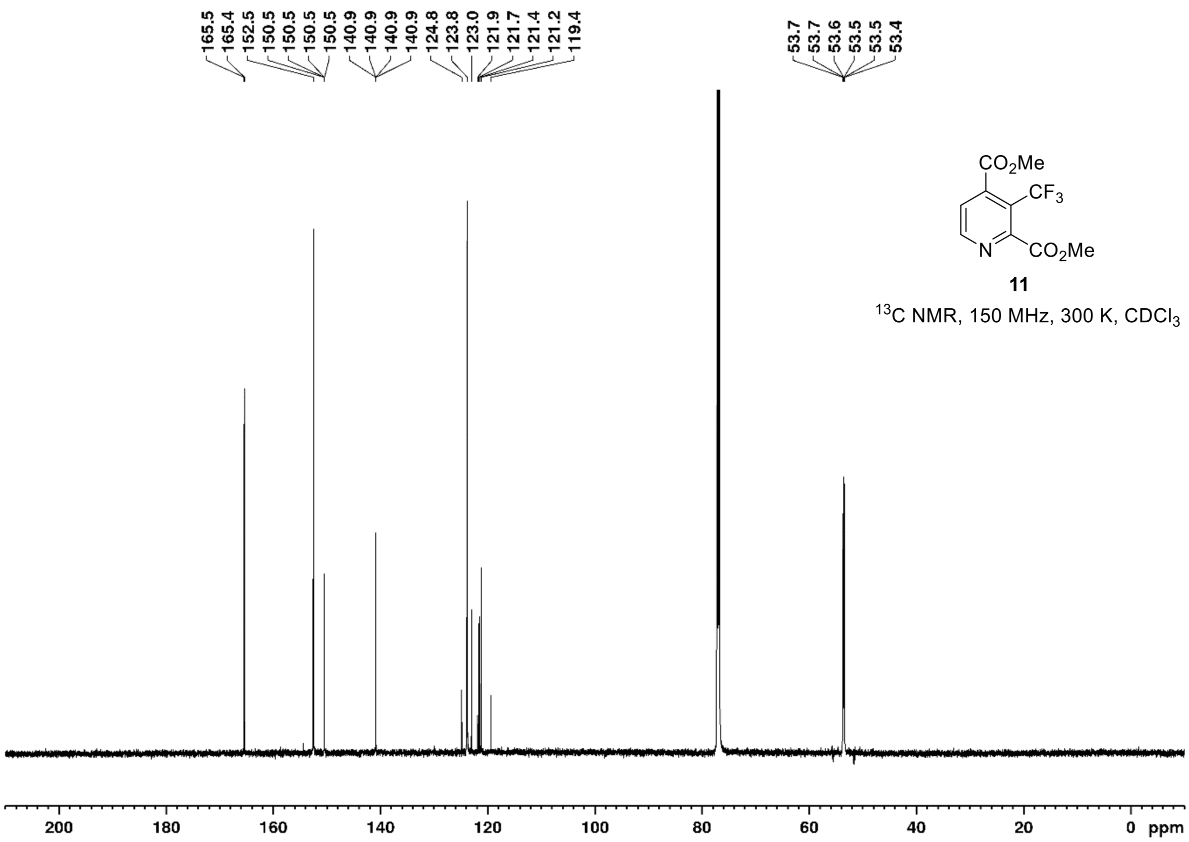
165.5
165.4
152.5
150.5
150.5
150.5
150.5
140.9
140.9
140.9
140.9
124.8
123.8
123.0
121.9
121.7
121.4
121.2
119.4
53.7
53.7
53.6
53.5
53.5
53.4
CO2Me
CF3
N
CO2Me
11
13C NMR, 150 MHz, 300 K, CDCl3
200 180 160 140 120 100 80 60 40 20 0 ppm

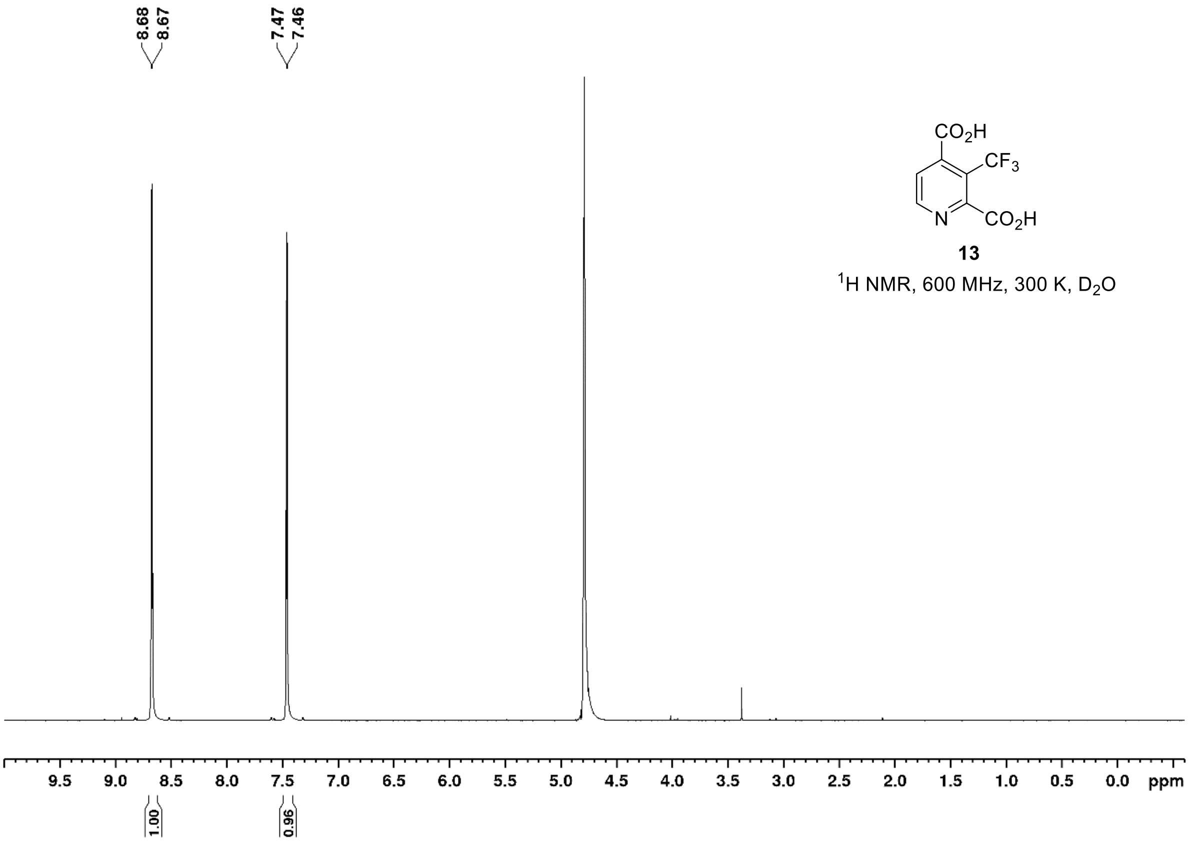
8.68
8.67
7.47
7.46
CO2H
CF3
N
CO2H
13
1H NMR, 600 MHz, 300 K, D2O
9.5 9.0 8.5 8.0 7.5 7.0 6.5 6.0 5.5 5.0 4.5 4.0 3.5 3.0 2.5 2.0 1.5 1.0 0.5 0.0 ppm
1.00
0.96

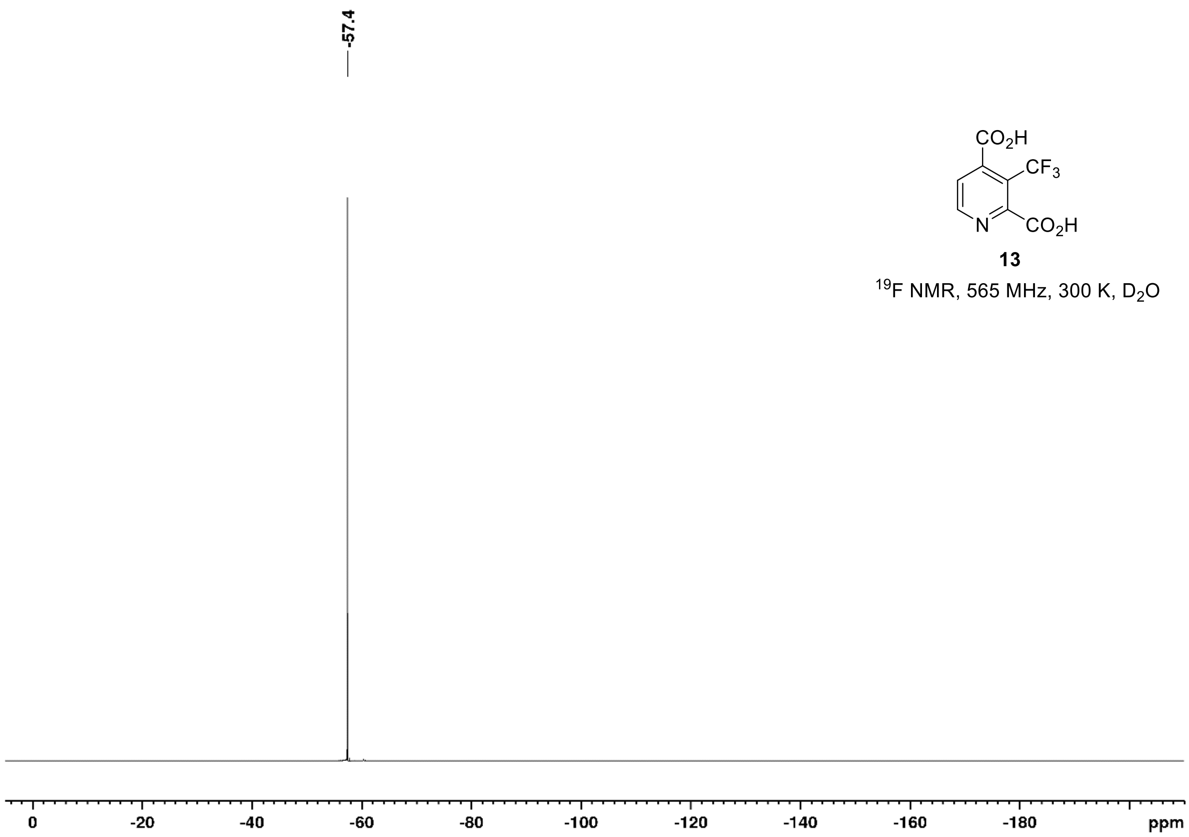

13

^{19}F NMR, 565 MHz, 300 K, D_2O

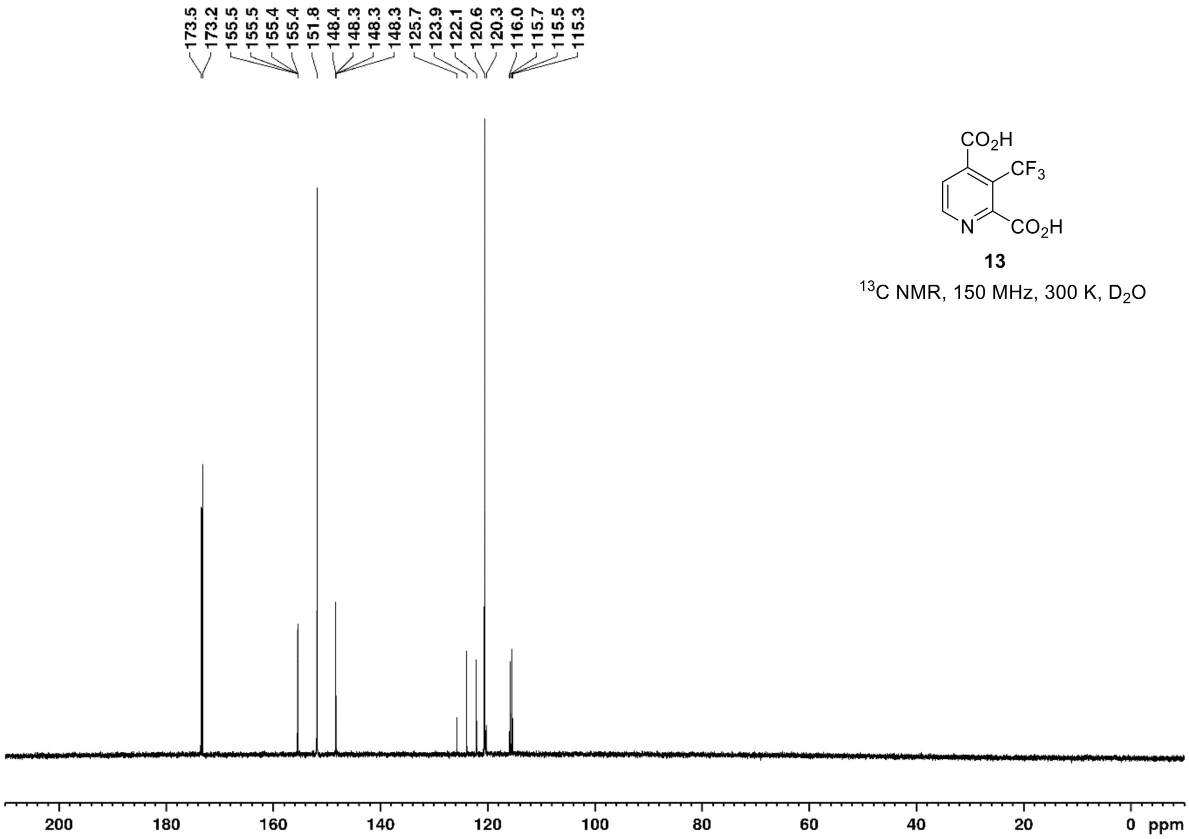

13

^{13}C NMR, 150 MHz, 300 K, D_2O

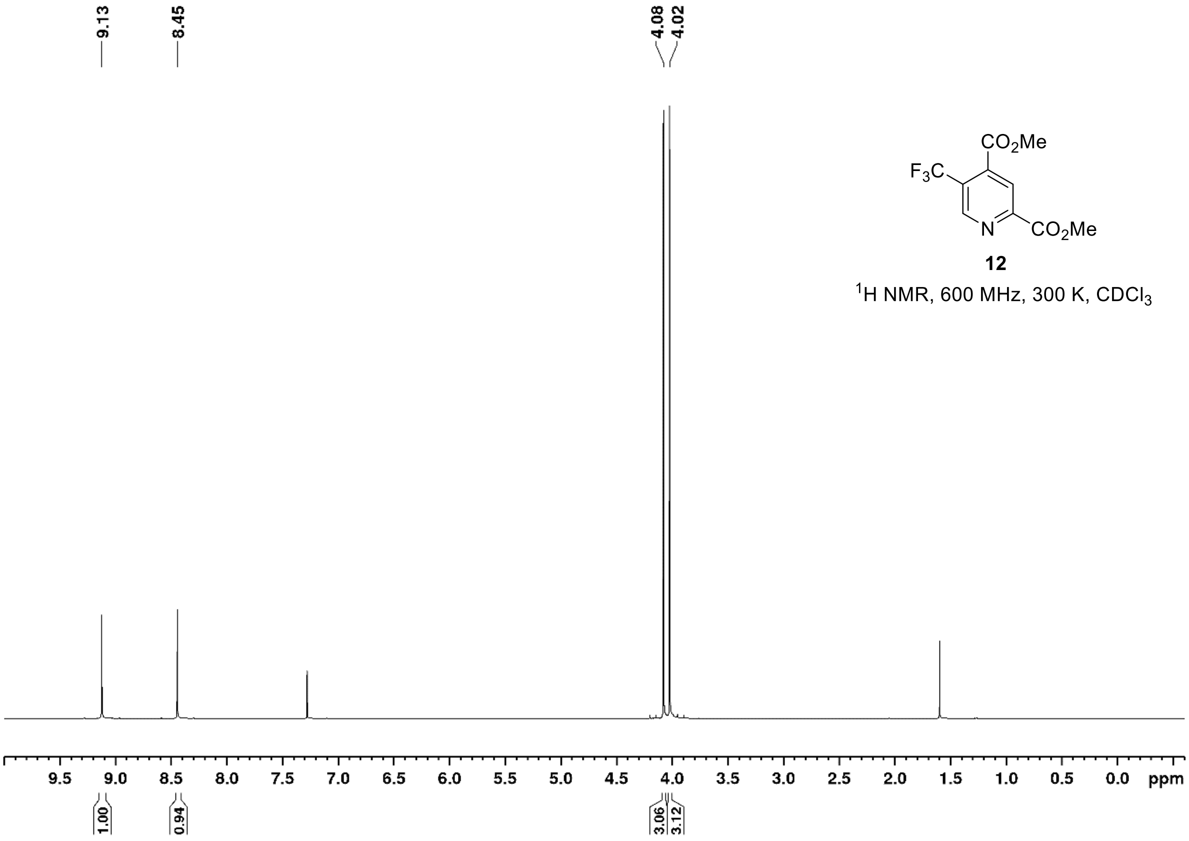

12

^{1}H NMR, 600 MHz, 300 K, $CDCl_3$

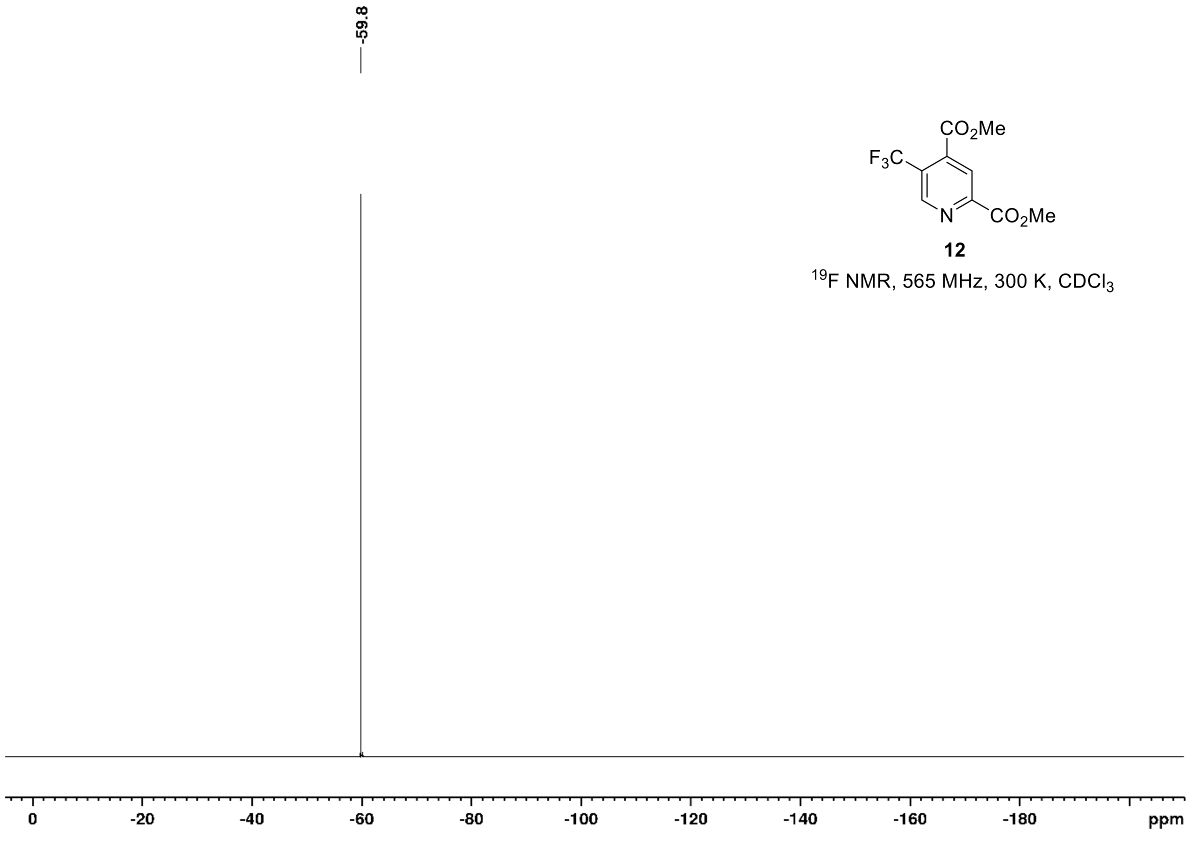

12

^{19}F NMR, 565 MHz, 300 K, $CDCl_3$

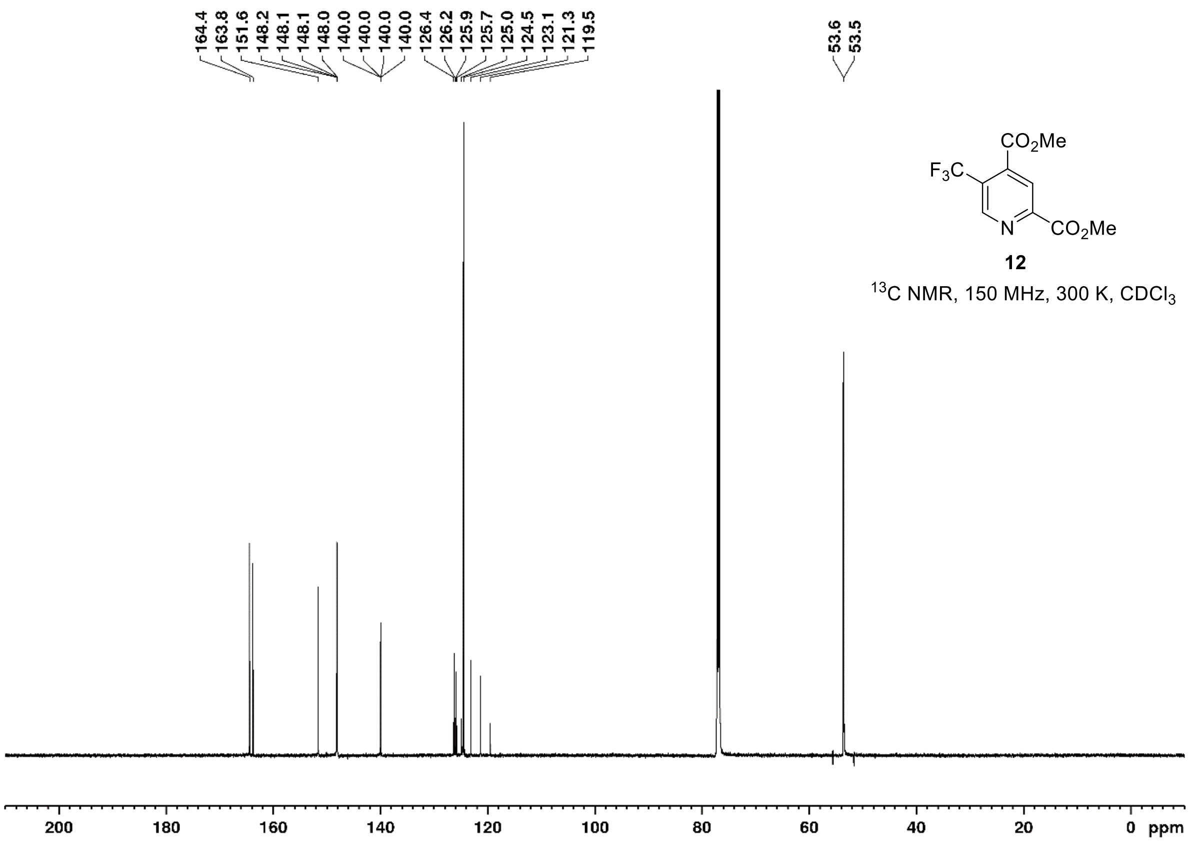
164.4
163.8
151.6
148.2
148.1
148.1
148.0
140.0
140.0
140.0
140.0
126.4
126.2
125.9
125.7
125.0
124.5
123.1
121.3
119.5
53.6
53.5
CO2Me
F3C
N
CO2Me
12
13C NMR, 150 MHz, 300 K, CDCl3
200
180
160
140
120
100
80
60
40
20
0
ppm

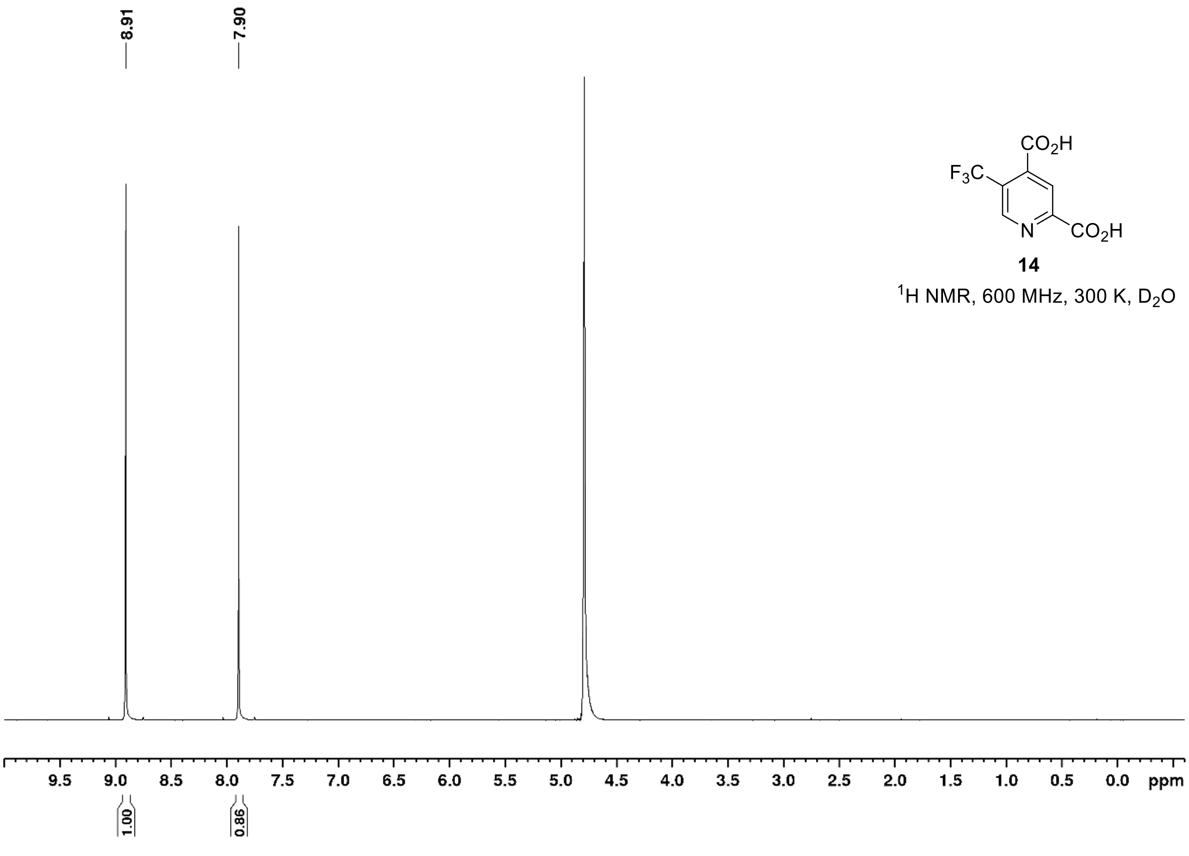
8.91
7.90
CO2H
F3C
N
CO2H
14
1H NMR, 600 MHz, 300 K, D2O
9.5
9.0
8.5
8.0
7.5
7.0
6.5
6.0
5.5
5.0
4.5
4.0
3.5
3.0
2.5
2.0
1.5
1.0
0.5
0.0
ppm
1.00
0.86

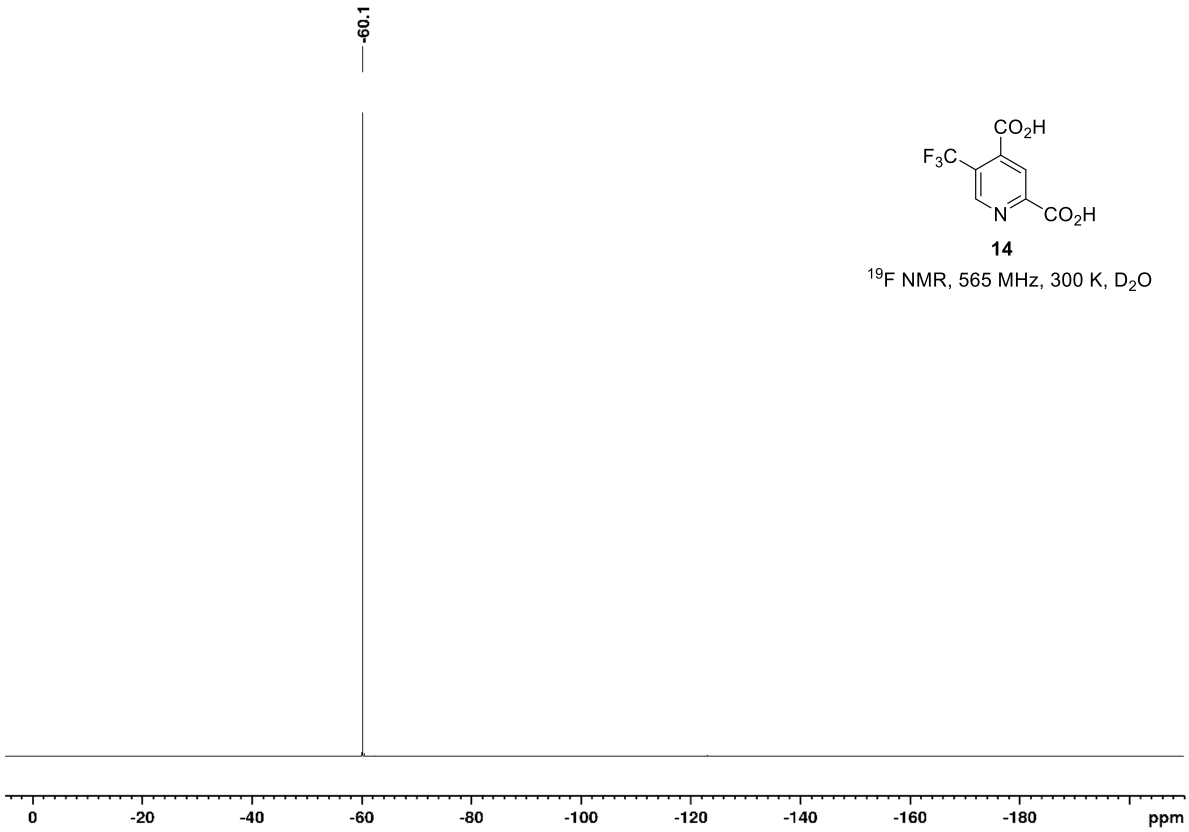

14

^{19}F NMR, 565 MHz, 300 K, D_2O

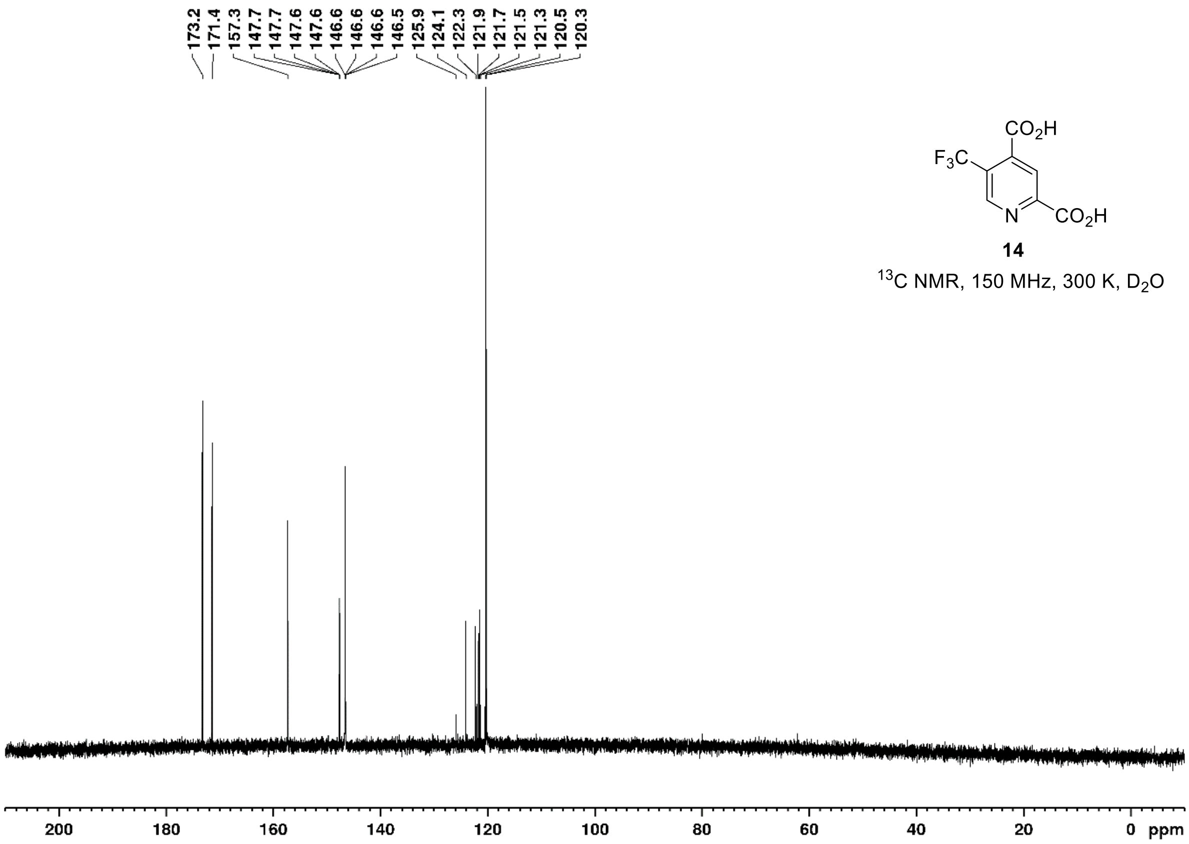

14

^{13}C NMR, 150 MHz, 300 K, D_2O
